# ﻿A new species of Anemonefish from French Polynesia, *Amphiprionmaohiensis*, (Pomacentridae, Amphiprioninae), the Polynesian anemonefish

**DOI:** 10.3897/zookeys.1244.141409

**Published:** 2025-07-10

**Authors:** James L. O’Donnell, Ricardo Beldade, Jason Johns, Giacomo Bernardi

**Affiliations:** 1 Department of Ecology and Evolutionary Biology, University of California Santa Cruz, 115 McAllister Way, Santa Cruz, CA 95060, USA; 2 712 S. Eureka St., Redlands, CA, 9237, USA; 3 Facultad de Ciencias Biológicas, Universidad Católica de Chile, 340 Av Bernardo O’Higgins, Santiago, Chile

**Keywords:** *Amphiprionmaohiensis*, cryptic speciation, French Polynesia, Polynesian anemonefish

## Abstract

Anemonefishes (Teleostei, Pomacentridae) comprise approximately 28 species of damselfishes that exclusively live symbiotically with sea anemones. Distribution ranges vary, with some species only found in few isolated islands and others with ranges that span almost the entire Indo-Pacific. The orange-fin anemonefish, *Amphiprionchrysopterus* shows a wide distribution, from Australia to French Polynesia, extending north to Micronesia and the Marshall Islands. Two main color morphs exist, with a morph that displays a white tail and a second morph that has an orange tail. In French Polynesia, only the orange-tail morph is present, while in the rest of the range, the white-tail morph is prevalent, while the orange-tail morph is also present, but very uncommon. Here we assessed the potential presence of a cryptic species in French Polynesia based on morphology, mitochondrial markers, and full genome sequencing. Morphological and genetic results were consistent in identifying a separate group in French Polynesia, which we describe here as a new species: the Polynesian anemonefish, *Amphiprionmaohiensis*.

## ﻿Introduction

The anemonefishes are a monophyletic clade comprised of 28 species in the genus *Amphiprion* (Tang et al. 2021; Marcionetti et al. 2024; Schmid et al. 2024). These damselfishes (Pomacentridae, Amphiprioninae) are obligate mutualists that live amongst the tentacles of sea anemones on coral reefs of the Indian and Pacific oceans (Titus et al. 2024). While their conspicuous behavior makes them a favorite among divers and photographers, the group is under active taxonomic revision. Most recently, the monotypic *Premnasbiaculeatus* was reassigned to *Amphiprionbiaculeatus* (Tang et al. 2021), and two new cryptic species were described (Allen et al. 2008, 2010), both regional endemics of the southwestern Pacific, a region with a traditionally underestimated rate of endemism (Drew et al. 2008).

**Figure 1.**
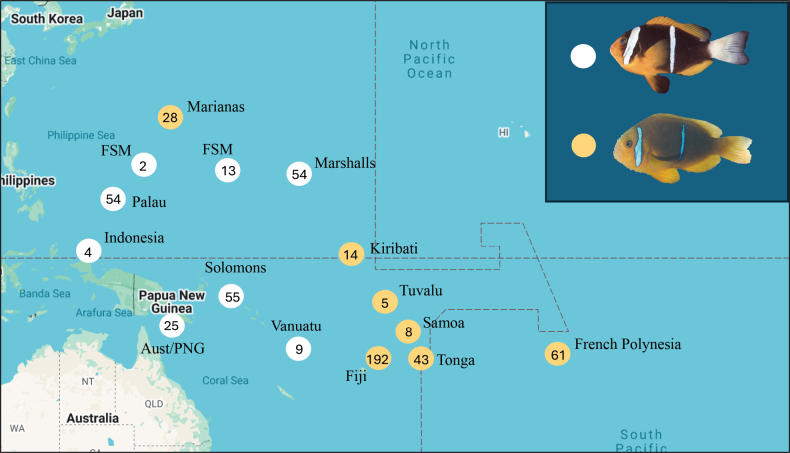
Distribution map of *Amphiprionchrysopterus* species complex (*A.chrysopterus* in the west, *A.maohiensis* in French Polynesia). Localities on the map refer to places used in this study. Localities with ‘white tail’ coloration are shown with white circles, while the typical ‘orange tail’ is shown as orange circles. Numbers inside the circles represent the number of individuals that were scored, in those areas, based on iNaturalist pictures. Fish pictures are from Palau (neotype-BPBM 6906, Bishop Museum, picture credit John Randall/FishBase), and Moorea, French Polynesia (picture credit Giacomo Bernardi).

Range distribution varies widely across the genus. Some anemonefish species display very restricted ranges, such as the white snout anemonefish, *Amphiprionmccullochi*, which is only found on Lord Howe Island and adjacent islets. Other species have very broad ranges, including *A.clarkii*, which is found throughout the Indian and Pacific Oceans. The orange-fin anemonefish, *Amphiprionchrysopterus*, has among the largest ranges of the anemonefishes in the Pacific, occurring in the Marianas, the Federated States of Micronesia, Papua New Guinea, and the Great Barrier Reef eastward to French Polynesia (He et al. 2018) (Fig. 1). The species was described by Cuvier (Cuvier and Valenciennes 1830) based on drawings made by Russian naturalists during their 1819–1821 circumglobal Vostok expedition, with no locality information. The name was upheld in Allen’s comprehensive 1972 work in the interest of parsimony, and a specimen collected from Palau (BPBM 6906) was designated as the neotype (Fig. 1) (Allen 1972).

Despite this large contiguous range, notable differences in coloration between individuals in the eastern (loosely defined here as Fiji and eastward) and western extents of the range are present, suggesting a potential for genetic differences and cryptic speciation. The main difference is in the caudal region, where individuals in the eastern part of the range have an orange caudal fin and caudal peduncle, while most of those in the western part of the range have white caudal fin and caudal peduncle (Fig. 1). Because subtle color differences among reef fish populations can reflect real species’ boundaries (Bernardi et al. 2002), we investigated the extent to which these geographically separated color morphs represent distinct evolutionary lineages, with a specific interest in the French Polynesian individuals.

We analyzed data regarding the potential distinctness of the eastern and western morphs from three sources: first, we examined the morphology of museum and collected specimens of *A.chrysopterus* from across the documented range. Second, we made observations of the coloration of individuals in the field and capitalized on georeferenced pictures available online. Lastly, we conducted a phylogenetic analysis of individuals from across the species’ range using mitochondrial markers and full nuclear genome resequencing. Taken together, these data indicate the presence of a new species in French Polynesia that we describe below.

## ﻿Methods

### ﻿Sampling and collections

Samples were collected using hand nets while free or scuba diving: *Amphiprionmaohiensis* in Moorea (French Polynesia), *Amphiprionchrysopterus* at Ulithi Atoll (Federated States of Micronesia), Kimberly (Papua New Guinea), and the Solomon Islands (Table 1). In addition, we collected samples of *A.sandarocinos* in Papua New Guinea and *A.perideraion* Busuanga (Philippines) and Ulithi Atoll as outgroups (Table 1). Specimens were then preserved in 95% ethanol and kept at −20 °C when back at the laboratory.

**Table 1.** Samples references and collection sites.

Species	Specimen	Figure legend	Locale
*A.maohiensis*	CAS202634A	FP_202634a	French Polynesia, Tuamotus
	CAS248601	FP_MOO1	French Polynesia, Moorea
	CAS248602	FP_MOO2	French Polynesia, Moorea
		AMCP_059_MOO	French Polynesia, Moorea
		AMCP_060_MOO	French Polynesia, Moorea
		MOO_73	French Polynesia, Moorea
		MOO_071201	French Polynesia, Moorea
*A.chrysopterus*	CAS7418A	FIJ_7418a	Fiji
	CAS7418B	FIJ_7418b	Fiji
		AMCP_001_Fij	Fiji
	CAS208514a	PAL_208514a	Palau
	CAS208514b	PAL_208514b	Palau
	CAS202579a	PAL_202579a	Palau
	CAS202579b	PAL_202579b	Palau
	CAS208508	PAL_208508	Palau
	CAS202638	PAL_202638	Palau
	CAS233687a	PAL_233687a	Palau
	CAS233687b	PAL_233687b	Palau
	CAS202636	PAL_202636	Palau
	CAS202641	PAL_202641	Palau
	CAS208509	PAL_208509	Palau
	CAS208510	PAL_208510	Palau
	CAS208513	PAL_208513	Palau
		AMCP_HKN219_PNG	Papua New Guinea
		AMCP_001_SOL	Solomon Islands
		UAR_012401	Micronesia, Ulithi, Falalop
		UAR_012402	Micronesia, Ulithi, Falalop
		UAR_012403	Micronesia, Ulithi, Falalop
		UAR_012404	Micronesia, Ulithi, Falalop
		FMH_012401	Micronesia, Ulithi, Falalop
		FMH_012402	Micronesia, Ulithi, Falalop
		LOO_022401	Micronesia, Ulithi, Loos’yep
*A.sandarocinos*		AMSA_USU016	Philippines
*A.perideraion*		AMPI_USU007	Philippines

### ﻿Comparative material examined, morphology and coloration

We examined preserved individuals at the California Academy of Science, from Fiji, Palau, and French Polynesia, as well as individuals that we collected from French Polynesia (Table 1). Measurements and counts follow Allen (1972) and are expressed as percent standard length. Measurements were made to the nearest millimeter. Morphological differences were estimated using Principal Component Analyses that were plotted and visualized using the R packages dyplr and ggplot2 (Wickham 2016; Wickham et al. 2023). Coloration differences of the caudal fin were examined by looking at georeferenced pictures obtained by iNaturalist (https://www.inaturalist.org, accessed 04/2025).

### ﻿Genetics

Genomic DNA was extracted from fin tissue using a phenol-chloroform protocol (Sambrook 1989). DNA was then used to amplify a mitochondrial gene to compare it with sequences from the literature and also used to sequence full genomes.

Amplification by PCR of the cytochrome oxidase subunit 1 (COI) region was carried out using the primers and parameters described by Bernardi and Crane (1999). In addition, for samples where full genomes were sequenced (described below), we extracted the complete mitochondrial genome by aligning the genomic data to the complete mitochondrial genome of *Amphiprionclarkii* (GenBank NC_023967) using Geneious. The cytochrome oxidase region was then extracted from the full mitochondrial genomes. Similar COI sequences were then obtained from GenBank using BLAST, and aligned using Geneious (Kearse et al. 2012). Phylogenetic trees were obtained using the R package APE (Paradis and Schliep 2019) based on the neighbor-joining method using the best fitting substitution model as determined using ModelTest in APE.

### ﻿Genomics

Whole genome libraries were prepared with NEBNext Ultra II FS DNA Library Prep Kit for Illumina and sequenced at the QB3 Genomics facility at the University of California Berkeley. Variants calling was performed using SNP-Archer (Mirchandani et al. 2024). To explore genetic differentiation via principal component analysis (PCA), we filtered the complete SNP dataset for sites with minor allele frequency >0.05 and less than 10% missing genotypes, then further pruned this set to randomly select SNPs separated by 1 kb or more with bcftools + prune -n 1 -N rand -w 1 kb. This pruning window size was intended to speed up downstream computation. Following this, we calculated PCAs at the whole genome level and at genomic regions of interest using the plink v1.90b7 function –pca (Purcell et al. 2007). We also estimated genetic distances between individuals using plink function – distance square, which allowed for the phylogenetic inference that was obtained using the R package APE (Paradis and Schliep 2019).

## ﻿Results

### ﻿Coloration

**Figure 2.**
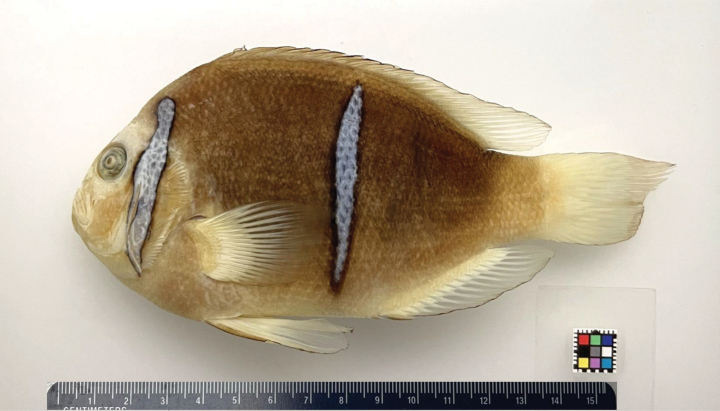
Holotype of *Amphiprionmaohiensis*, Polynesian anemonefish (CAS-ICH 248601, adult, Opunohu, Moorea, French Polynesia, 17.489°S, 149.8668°W, collected with a hand net by O’Donnell, J., 21 June 2016. Photo credit: Shinji Yamamoto.

All samples that we observed in French Polynesia have an orange caudal peduncle and caudal fin (Figs 1, 2). In contrast, samples we observed in the western region of the range: Solomon Islands, Papua New Guinea, and Micronesia, had a white caudal peduncle and caudal fin. Yet, in that region, some individuals from Micronesia have been reported to have the orange coloration as well (Myers 1991). Georeferenced pictures that we analyzed from iNaturalist included 880 records (accessed 04/2025). Some pictures contained multiple individuals, other pictures only showed the front part of the body (usually the hind part of the body being hidden by the tentacles of the host anemone). This dataset resulted in 565 individuals where the color of the caudal fin could be scored (Fig. 1). The color of the caudal fin was either white or orange at a given site, with no site that had both color types except for three individuals (assuming that the georeferenced pictures have the correct coordinates). In the Solomon Islands, where individuals have white caudal fins, one individual had a black caudal fin, and one individual had an orangish caudal fin. In Vanuatu, where individuals have white caudal fins, one individual had an orange caudal fin. Of the remaining 562 scored individuals, no other instance of sympatric tail colors was observed. As described in the Introduction, all individuals East of Fiji had an orange caudal fin (Fig. 1). Individuals west of that line had a white caudal fin, except for individuals in the Marianas, where all individuals had an orange caudal fin (Fig. 1).

### ﻿Morphology

**Figure 3.**
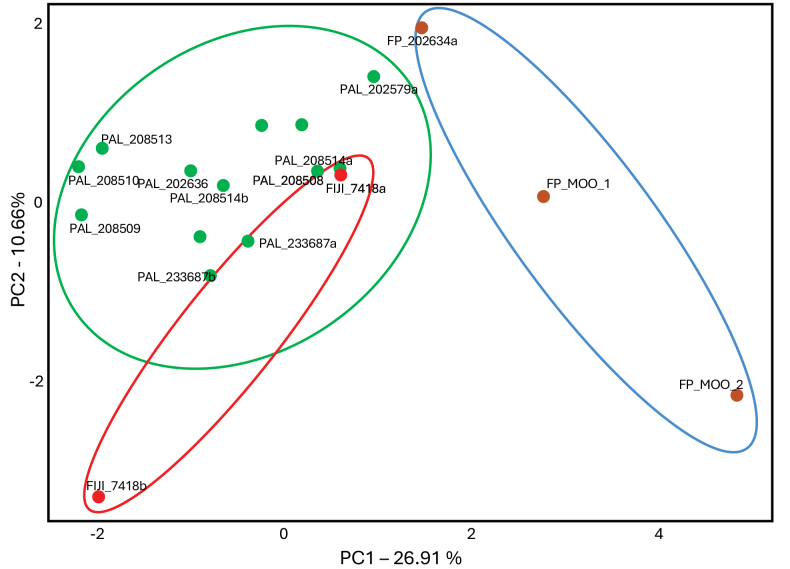
Principal components analysis (PCA) cluster plot of *Amphiprionchrysopterus* and *Amphiprionmaohiensis* based on morphological characters were created in R using the DYPLR and GGPLOT2 packages. *Amphiprionchrysopterus* samples from Palau and Fiji are in green and red, respectively; *Amphiprionmaohiensis* samples from French Polynesia are in brown.

We based our analyses on 28 morphometric measurements and 22 meristic ones (Suppl. material 1: table S1). The morphological measurements that most strongly separated eastern and western individuals were the length of the dorsal-fin base/DFL, length of the anal-fin base/AFL, length of the pectoral fin/PCL, length of the longest dorsal ray/DRL, and the length of the longest anal ray/ARL (Suppl. material 2: fig. S1). Principal component analysis based on morphological measurements is shown in Fig. 3. Western individuals from Fiji and Palau individuals showed overlapping PCA envelopes, while individuals from French Polynesia completely separated from their western counterparts (Fig. 3).

### ﻿Mitochondrial sequences

**Figure 4.**
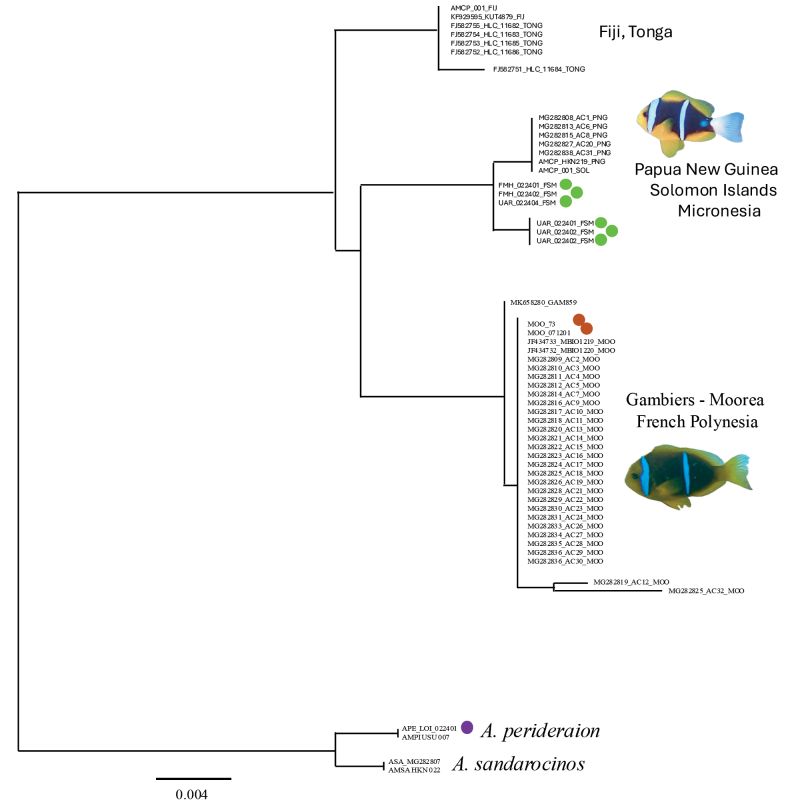
Phylogenetic relationships based on the mitochondrial cytochrome oxidase 1 (COI) region were obtained using the R package APE. Sequences of *Amphiprionchrysopterus* were obtained from Fiji, Tonga, Papua New Guinea, and the Solomon Islands. Sequences of *Amphiprionmaohiensis* were obtained from Moorea, French Polynesia. Outgroups included *Amphiprionperideraion* and *A.sandarocinos*. Acronyms represent the code name used in this paper (see Table 1), or the GenBank accession number, followed by three letters that indicate the collection locality. Sequences separate into three clade: one clade from Fiji and Tonga, one clade from Papua New Guinea, the Solomon Islands and Micronesia (picture of fish from Ulithi Atoll, Micronesia, picture credit, Nicole Crane), and one clade, that represents the new species, from French Polynesia (picture of fish from Moorea, French Polynesia, picture credit, Giacomo Bernardi). Some COI sequences were extracted from whole genome sequencing experiments shown on Fig. 5. Those sequences are labelled with colored circles that are also used on Fig. 5, for consistency and comparison.

We obtained mitochondrial COI sequences for three western individuals (Fiji, Papua New Guinea and Solomon Islands) and two eastern individuals (French Polynesia Moorea), as well as outgroup individuals (*A.perideraion* and *A.sandarocinos*). We also obtained mitochondrial sequences from the full genome experiments for individuals from Micronesia and French Polynesia. These data were supplemented with GenBank sequences from 10 western individuals, from Fiji, Tonga, and Papua New Guinea, and 29 eastern individuals from French Polynesia (Fig. 4).

Sequences of eastern and western pacific individuals separated into three monophyletic clades (Fig. 4). Sequences from French Polynesia (one individual from the Gambiers Archipelago, and all other individuals from Moorea) grouped into one monophyletic clade, while the western individuals separated into two clades; one clade included individuals from Fiji and Tonga, the other clade included individuals from Micronesia, the Solomons and Papua New Guinea. The status of those two western clades goes beyond the scope of this paper and description, but they might represent additional cryptic species.

### ﻿Sequence and time of divergence

The best substitution model based on ModelTest for our COI dataset was HKY. When using that model, we found that the divergence between the two outgroup species *A.perideraion* and *A.sandarocinos* was 0.6%. The divergence between the French Polynesia clade and the clade that includes Micronesian samples was 1.5%. When using conservative molecular clocks for fish COI regions (1% per My) (Guimarães et al. 2022), we estimated the time of divergence between these clades to be approximately 1.5 Mya. The divergence between two recognized species, *A.perideraion* and *A.sandarocinos*, is less than half that divergence in this dataset (0.6 Mya).

### ﻿Genomic sequences

**Figure 5.**
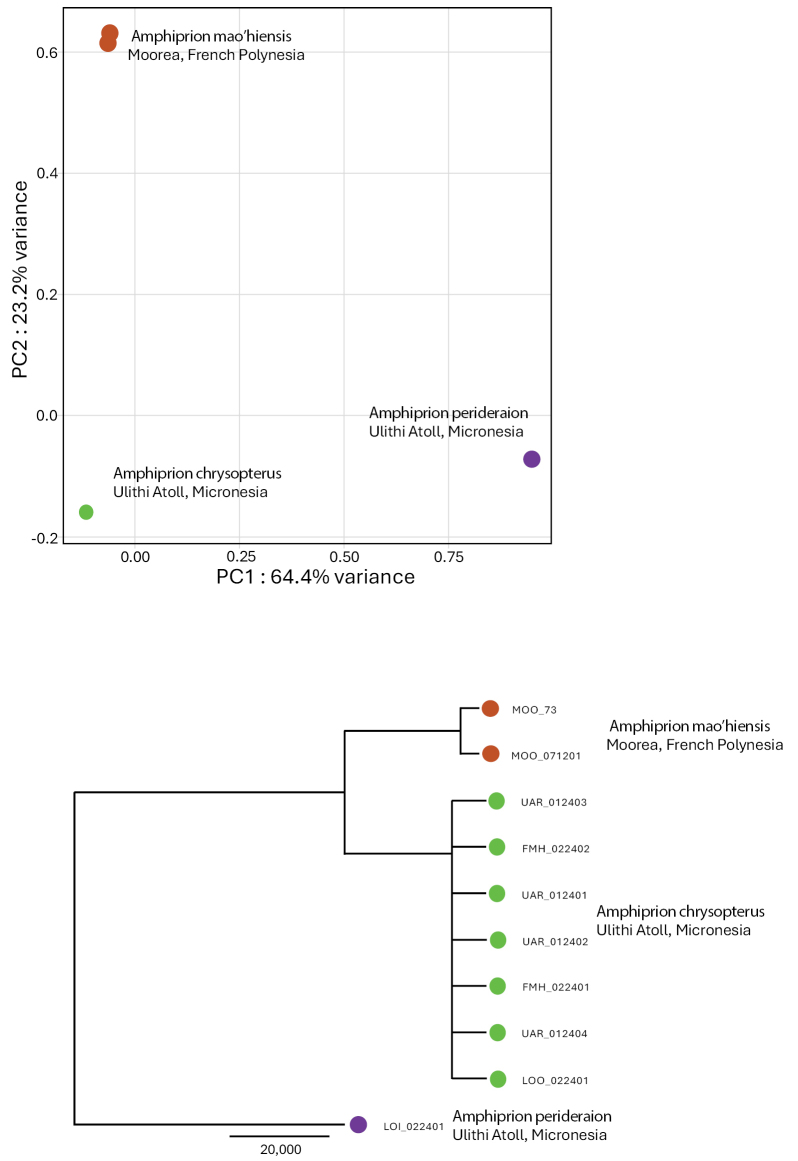
Genomics of *Amphiprionchrysopterus* and *Amphiprionmaohiensis*. The upper panel shows a Principal components analysis (PCA) cluster plot based on whole genome SNPs and created in R using the PLINK package. The lower panel shows the phylogenetic relationships of the same individuals as the upper panel. Genetic distances were obtained with PLINK and phylogenetic relationships were inferred using APE. Individuals of *Amphiprionmaohiensis* are from Moorea (brown), individuals of *Amphiprionchrysopterus* are from Ulithi Atoll, Micronesia (green), and the outgroup individual was *Amphiprionperideraion* from Ulithi Atoll, Micronesia (purple).

Whole genome sequences were obtained for two individuals from French Polynesia, eight individuals from Micronesia, and one outgroup individual (*Amphiprionperideraion*). Average genome coverage was 7.5×, and the filtering protocol we used resulted in 27.7 10^6^ SNPs. Genomic data were congruent with mitochondrial results. Indeed, both principal component analysis (PCA) and distance tree unambiguously separated western (Micronesia) and eastern (French Polynesia) individuals (Fig. 5), supporting the idea that these clades consistently differ both at the mitochondrial and nuclear DNA levels.

### ﻿Conclusion

The elevation to species level of the clade comprising individuals from French Polynesia rests on a convergence of evidence. Mitochondrial COI distinguishes this group as a monophyletic clade that is well separated from its close relatives (more than twice the genetic divergence between the closely related species *A.sandarocinos* and *A.perideraion*). Full genome sequencing is consistent with the mitochondrial data in separating French Polynesian individuals from individuals collected in Micronesia. Morphological differences were limited, but they also separated French Polynesian individuals. Caudal fin coloration was not found to be a distinctive character of the species, because orange caudal fin is found in individuals from Fiji and adjacent areas, as well as in the Marianas. Additional investigations are warranted to fully elucidate the status of populations in those areas.

## ﻿Taxonomy

### ﻿Polynesian anemonefish

#### ﻿ Amphiprion maohiensis

Taxon classificationAnimaliaPerciformesPomacentridae

O’Donnell, Beldade, Johns & Bernardi sp. nov.

ACA9C412-2F72-5C72-AB86-426A7AB8275C

https://zoobank.org/19B49601-3BE4-4E94-831D-F5F7F9F8FAF1

Figs 1 , 2 , 3 , 4 , 5 , Suppl. material 1: table S1

##### Polynesian Anemonefish.

***Type locality*.** Opunohu, Moorea, French Polynesia, 17.4889°S, 149.8668°W.

***Holotype*.** The holotype is preserved at the California Academy of Sciences, San Francisco, California, with the catalog number: CAS-ICH 248601, adult, Opunohu, Moorea, French Polynesia, 17.4889°S, 149.8668°W, collected with a hand net by O’Donnell, J., 21 June 2016 (Fig. 2).

***Paratypes*.** Two other individuals collected with the holotype (CAS-ICH 248602), one individual from same locality collected in September 2020 (CAS-ICH 248603), individuals from Raroia Atoll, Tuamotu Archipelago, French Polynesia, (CAS ICH 202634), and other individuals from French Polynesia (CAS-ICH 208511, CAS-ICH 208512 CAS-ICH 202578, and CAS-ICH 233689).

##### Comparative material.

*Amphiprionchrysopterus* CAS7418a, CAS7418a (Fiji), and CAS208514a, CAS208514b, CAS202579a, CAS202579b, CAS208508, CAS202638, CAS233687a, CAS233687b, CAS202636, CAS202641, CAS208509, CAS208510, CAS208513 (Palau).

##### Diagnosis and description.

A species of *Amphiprion*, distinguished by the following combination of characters: dorsal rays X–XI, 17; anal rays II, 14–15, tubed lateral line scales 35–40 (Table 1); adult coloration in life; iris dark; body, light orange to dark yellow; two white to bluish bars, the first behind the eye, the second midbody; all fins orange. Distinctive characters compared to *A.chrysopterus*, morphological: Dorsal fin length (>58% of standard length for *A.maohiensis*, <56% of standard length in *A.chrysopterus*), genetics: species can be unambiguously distinguished both with mitochondrial and nuclear genomic markers (reciprocally monophyletic clades).

##### Habitat.

Polynesian anemonefish usually occurs in association with *Radianthusmagnifica* (formerly *Heteractismagnifica*), the magnificent sea anemone, which is also by very far the most common sea anemone in French Polynesia. Less common sea anemone species may be found and may be associated with Polynesian anemonefish, indicating that they do not show an exclusive association with *R.magnifica*.

##### Etymology.

The term *ma’ohiensis* refers, in Polynesian, to the belonging of native land, ma’ohi. Apostrophes cannot be used in species names (International Code of Zoological Nomenclature, Article 32.5.2), and therefore the species name is *maohiensis*.

##### Common name.

We suggest Polynesian anemonefish as the common name of this species that is predominantly, if not exclusively, found in French Polynesia.

## Supplementary Material

XML Treatment for Amphiprion maohiensis

## ﻿Additional information

### Use of AI

No use of AI was reported.

### Author ORCIDs

Ricardo Beldade https://orcid.org/0000-0003-1911-0122

Jason Johns https://orcid.org/0009-0008-2381-8653

Giacomo Bernardi https://orcid.org/0000-0002-8249-4678

### Data availability

All of the data that support the findings of this study are available in the main text or Supplementary Information.

## Supplementary materials

Supplementary material 1 Distribution of morphological characters taken individually for *Amphiprionchrysopterus* (species 1) and *Amphiprionmaohiensis* (species 2) Data type pdf Explanation note Each character is labelled with its acronym that is described in Table 1. https://binary.pensoft.net/file/1371304This dataset is made available under the Open Database License (http://opendatacommons.org/licenses/odbl/1.0/). The Open Database License (ODbL) is a license agreement intended to allow users to freely share, modify, and use this Dataset while maintaining this same freedom for others, provided that the original source and author(s) are credited.James L. O’Donnell, Ricardo Beldade, Jason Johns, Giacomo Bernardi

Supplementary material 2 Samples references, collection sites, with morphological and meristic measurements Data type pdf Explanation note Standard length = STL, depth = D, width = W , head length = HL , snout length = SNL , eye = ED , interorbital = IO , caudal-peduncle depth = CPD , caudal-peduncle length = CPL , upper jaw length = UJL , predorsal length = PDL , preanal length = PAL , prepelvic length = PPL , length dorsal-fin base = DFL , length anal-fin base = AFL , length pectoral fin = PCL , length pelvic fin = PVL , length pelvic-fin spine = PVS , length first dorsal spine = DS1 , length second dorsal spine = DS2 , length third dorsal spine = DS3 , length last dorsal spine = DSL , length longest dorsal ray = DRL , length first anal spine = AS1 , length second anal spine = AS2 , length longest anal ray = ARL , length caudal fin = CFL , 1st bar width at contact of eye = B1W , 2nd bar width at LL = B2W , orsal spines = DS , dorsal soft rays = DR , dorsal rays total = Dtotal , anal spines = AS , anal soft rays = AR , anal rays total = Atotal , pelvic spines = PVS , pelvic soft rays = PVR , pelvic rays (hard, soft) = Pvtotal , caudal rays = CR , pectoral rays = PCR , scales in longitudinal series = SC , LL Scales = LLS , Scales below LL to origin of anal fin = LLB , scales above LL to middle of dorsal fin = LLA , lower operculum spines = LOS , upper operculum spines = UOS , preopercle spines = POS , suborbital spines = SOS , forehead scales = FHS , gill rakers on first branchial arch = B1E , 1st bar over dorsum = B1D , 2nd bar contacts dorsum = B2D , 3rd bar marking = B3. https://binary.pensoft.net/file/1371305This dataset is made available under the Open Database License (http://opendatacommons.org/licenses/odbl/1.0/). The Open Database License (ODbL) is a license agreement intended to allow users to freely share, modify, and use this Dataset while maintaining this same freedom for others, provided that the original source and author(s) are credited.James L. O’Donnell, Ricardo Beldade, Jason Johns, Giacomo Bernardi
