# Supplementary figures and images for: ﻿A new species of Anemonefish from French Polynesia, Amphiprionmaohiensis, (Pomacentridae, Amphiprioninae), the Polynesian anemonefish
Source: Zookeys. 2025 Jul 10;1244:225–37. doi: 10.3897/zookeys.1244.141409 (PMC12272074; doi:10.3897/zookeys.1244.141409)

# STL

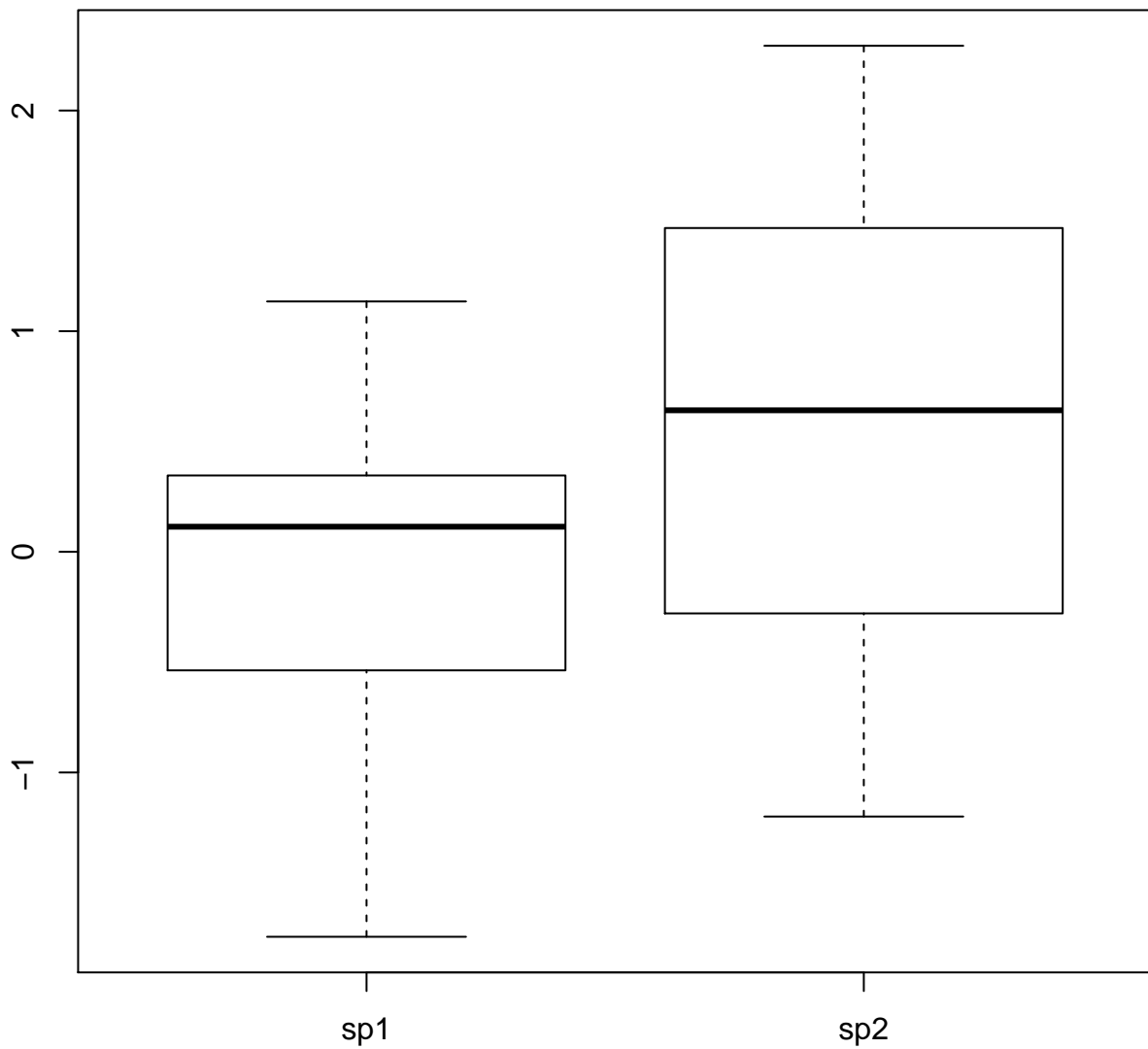

**D**

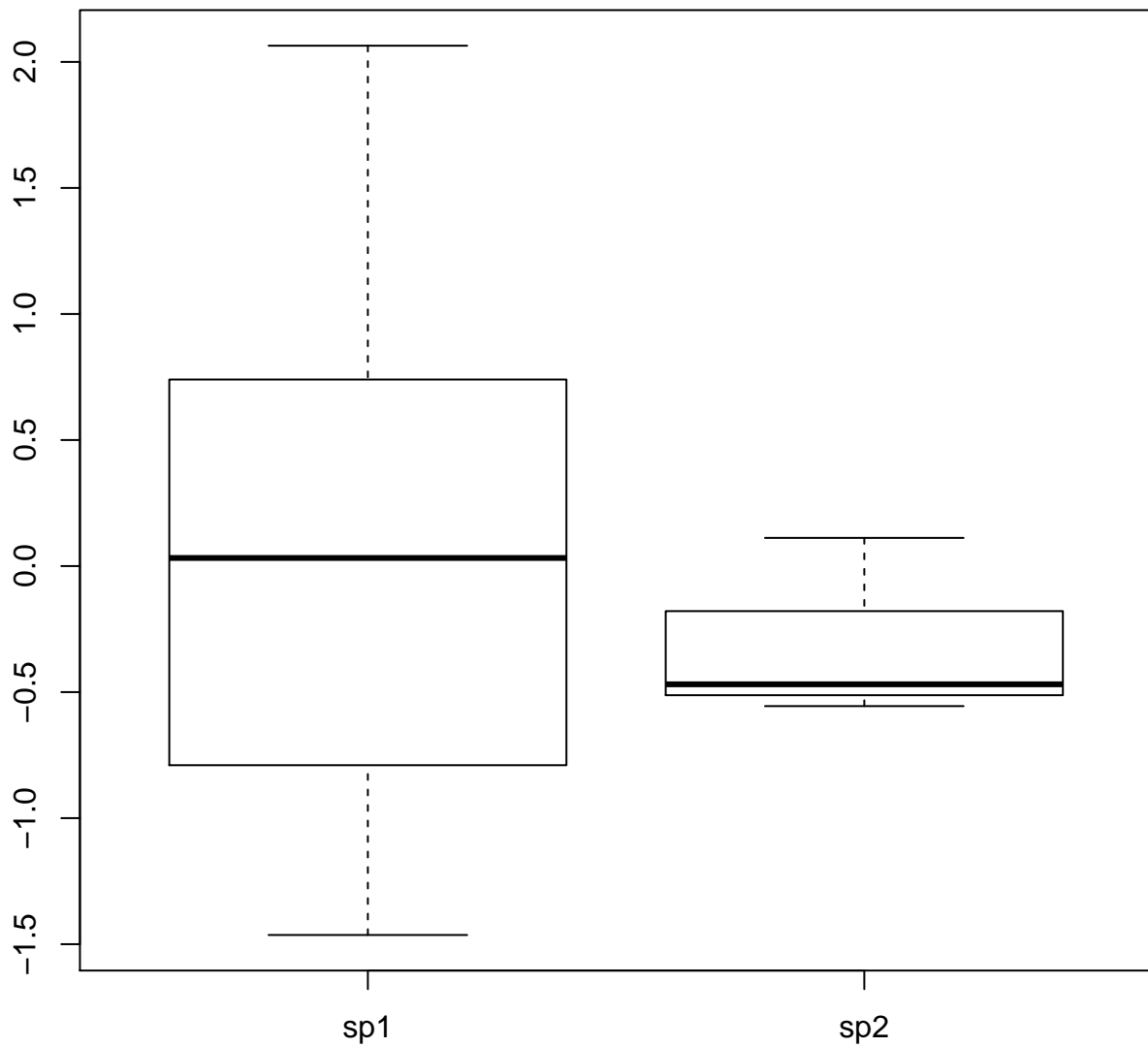

W

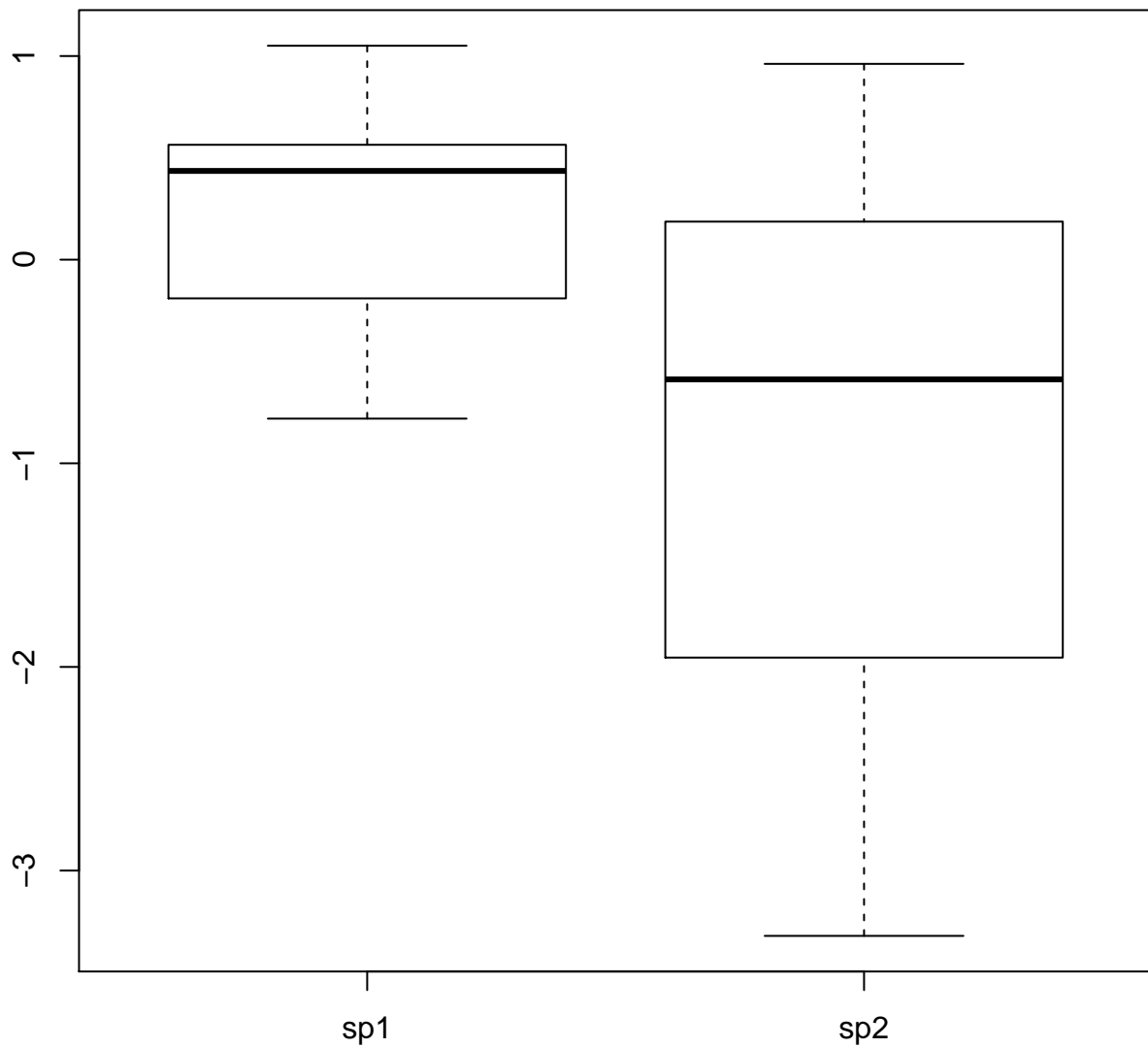

HL

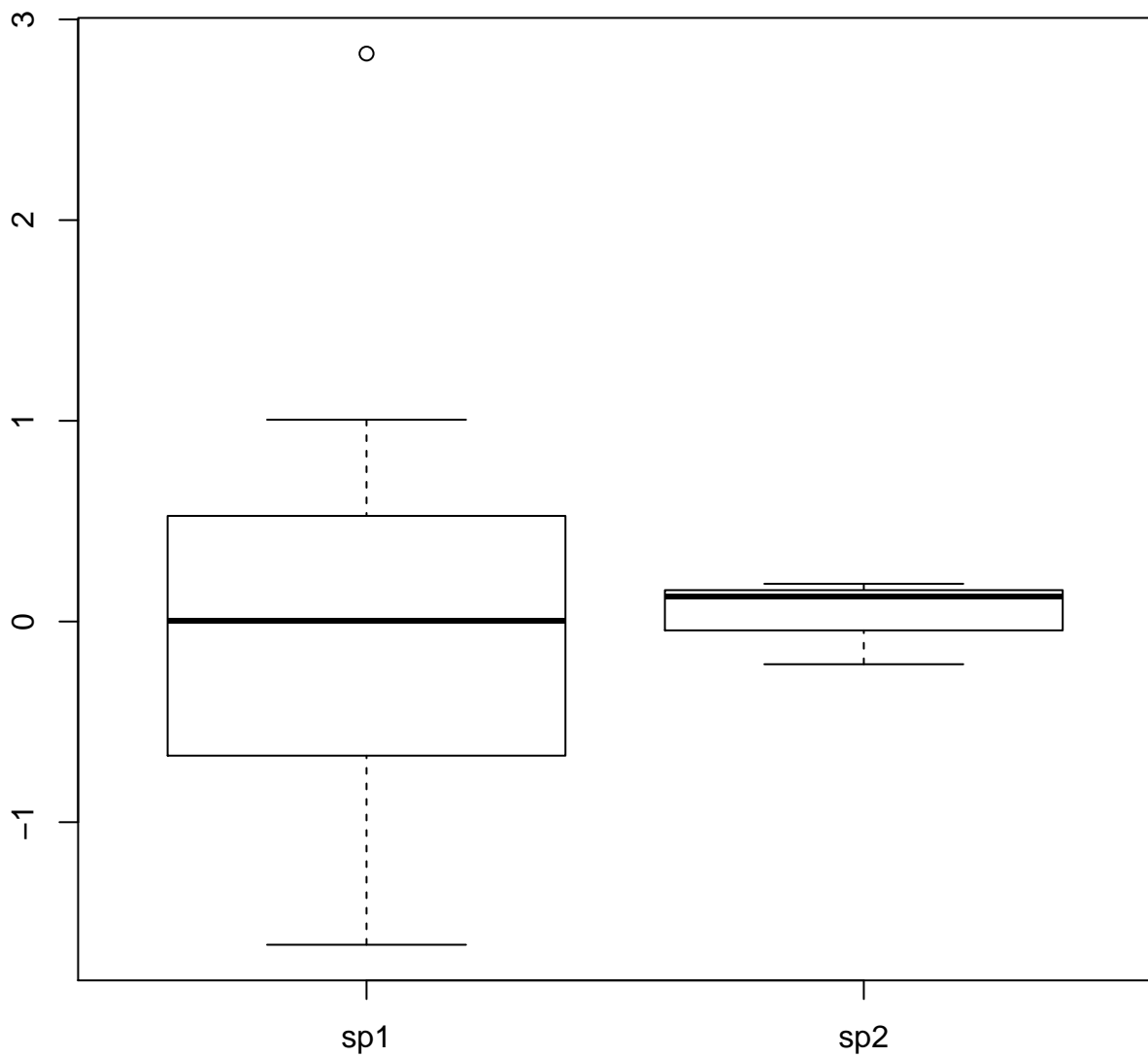

# SNL

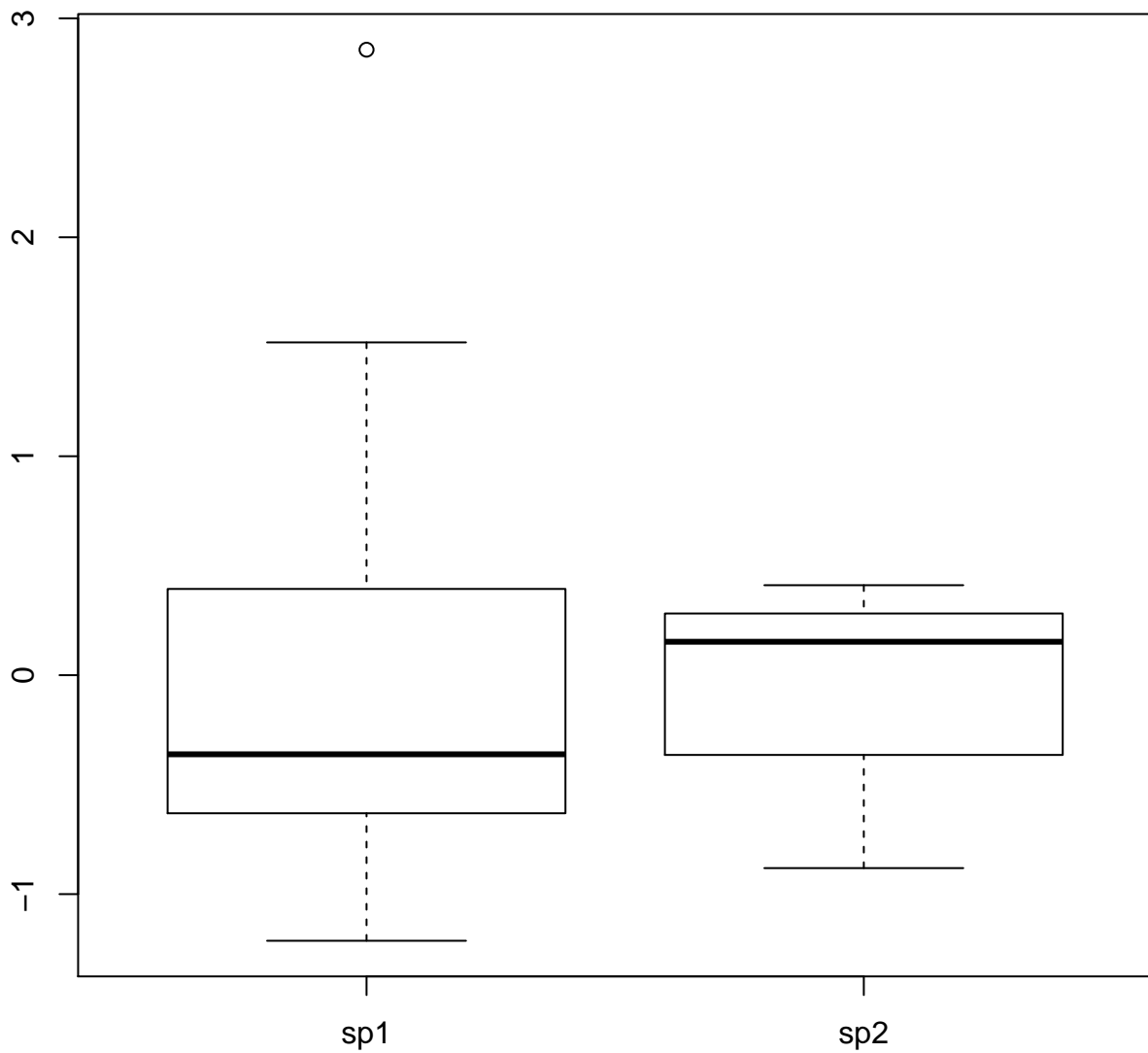

**ED**

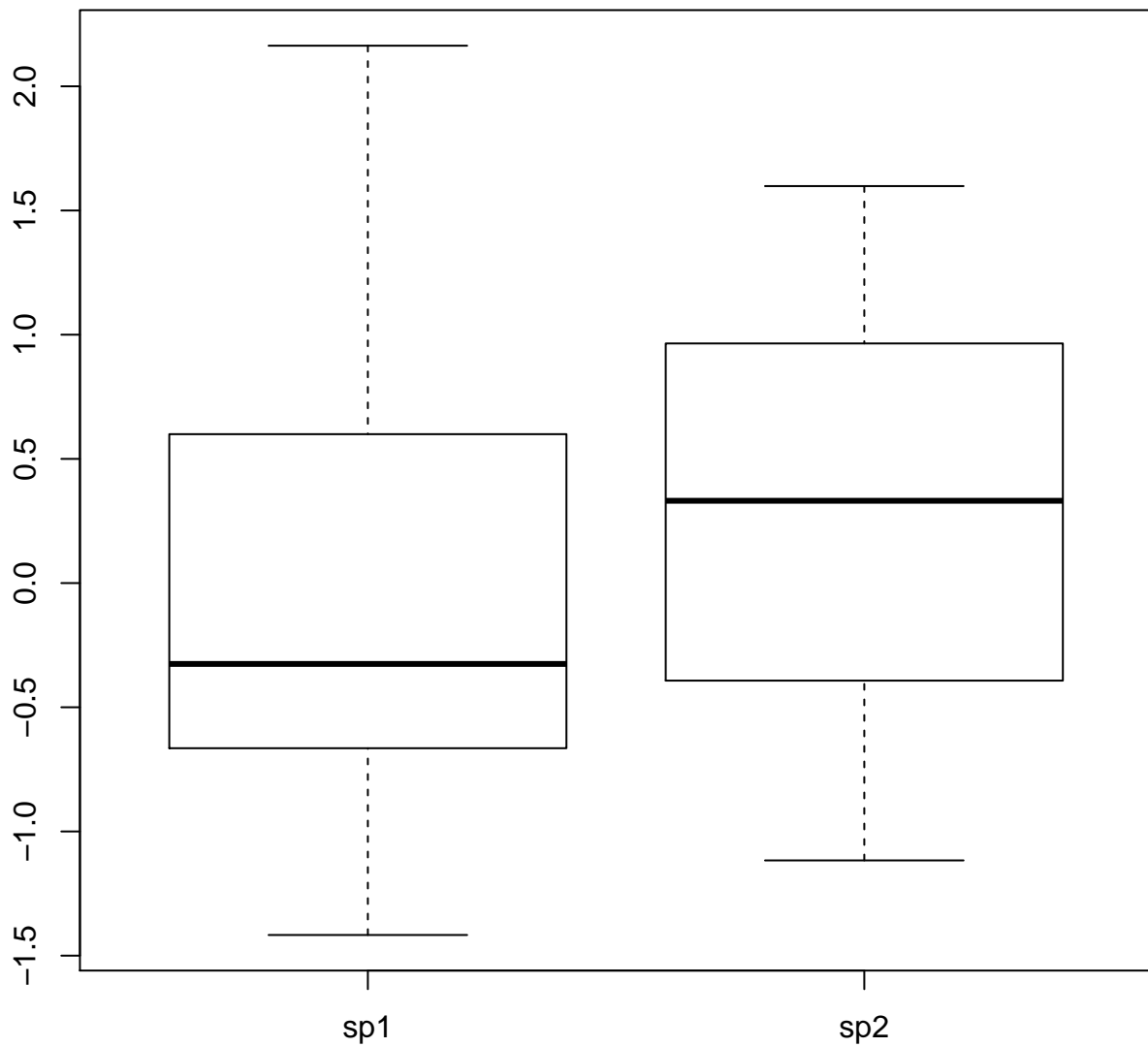

10

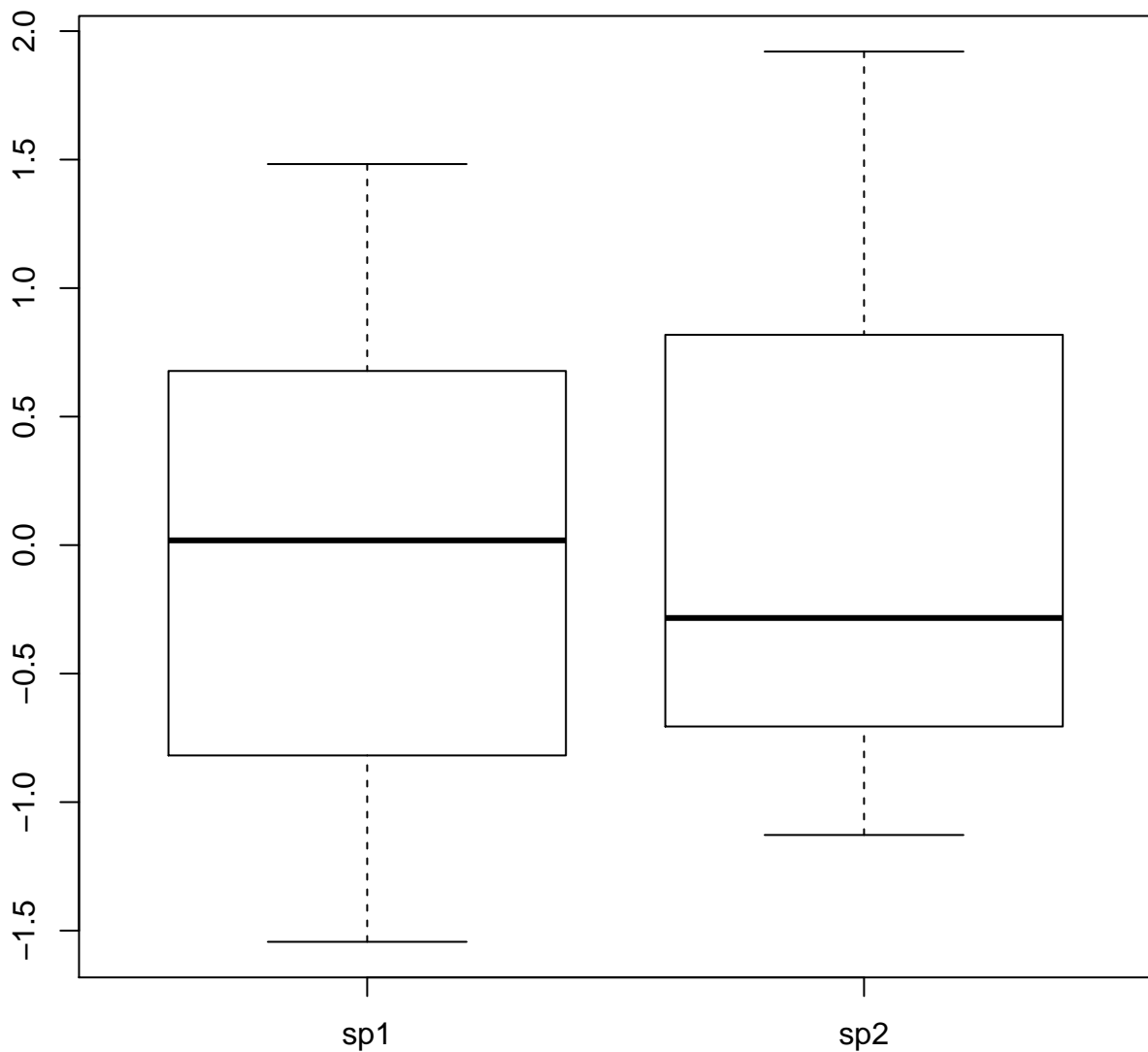

# CPD

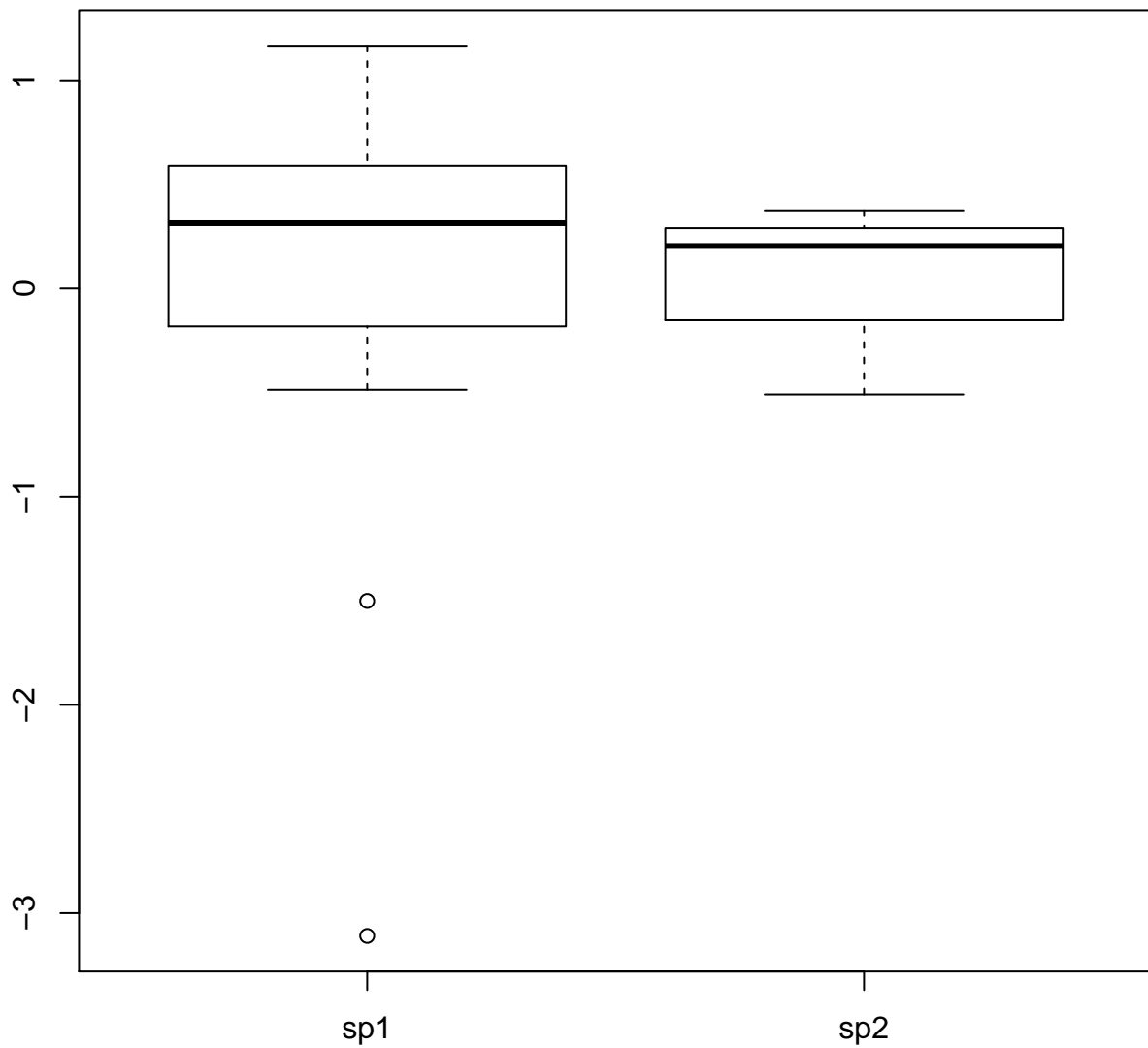

# CPL

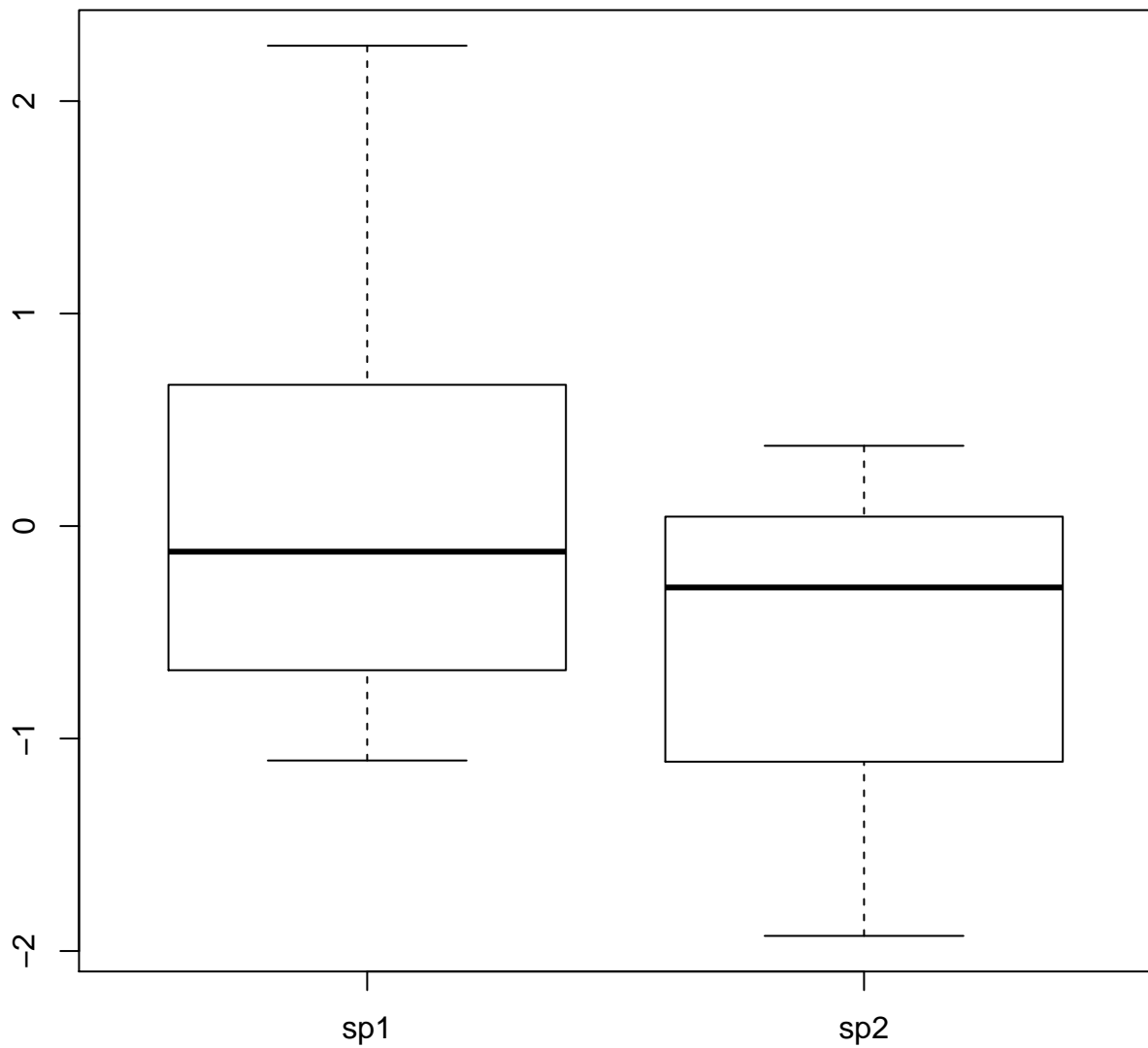

# UJL

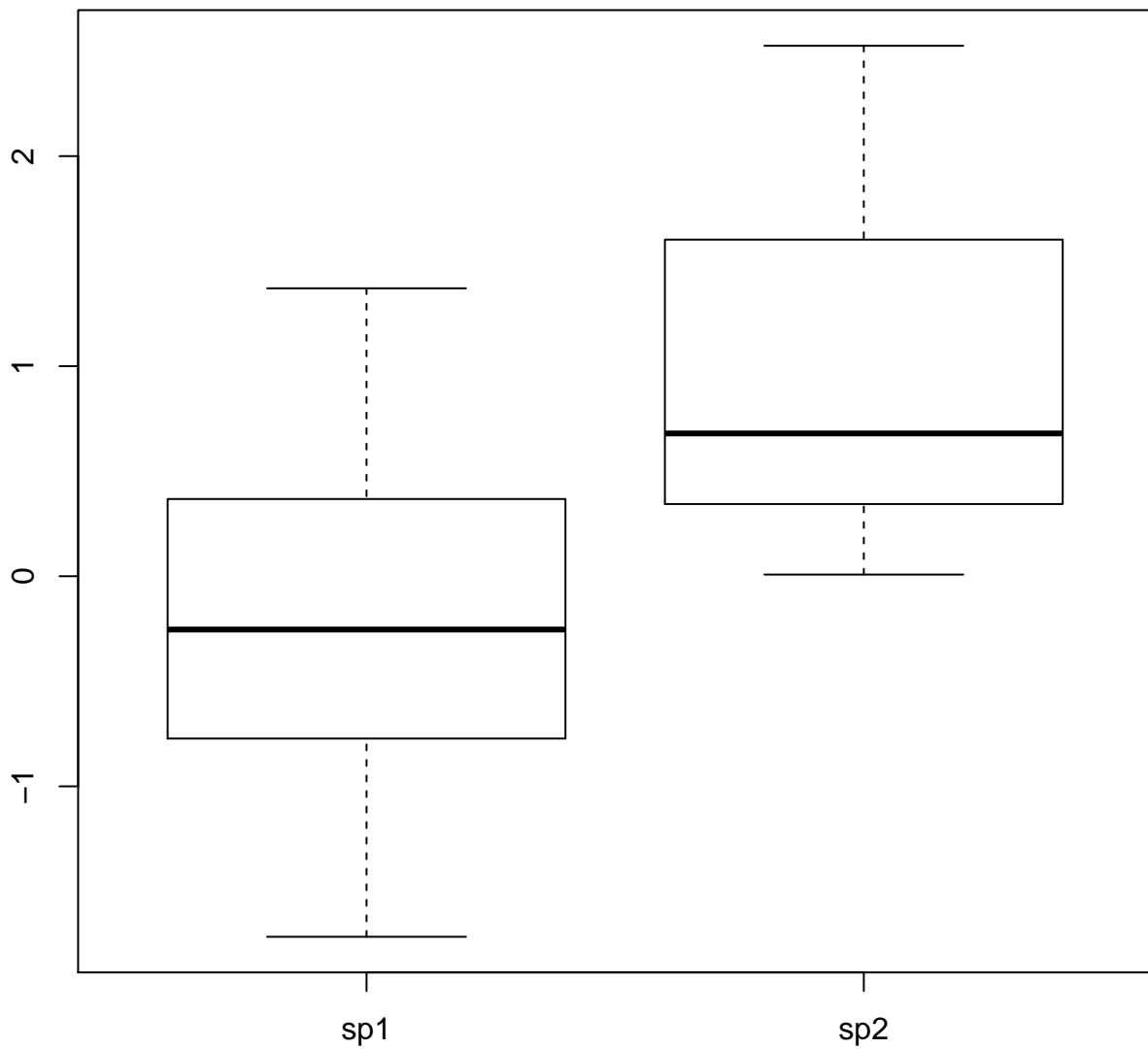

# PDL

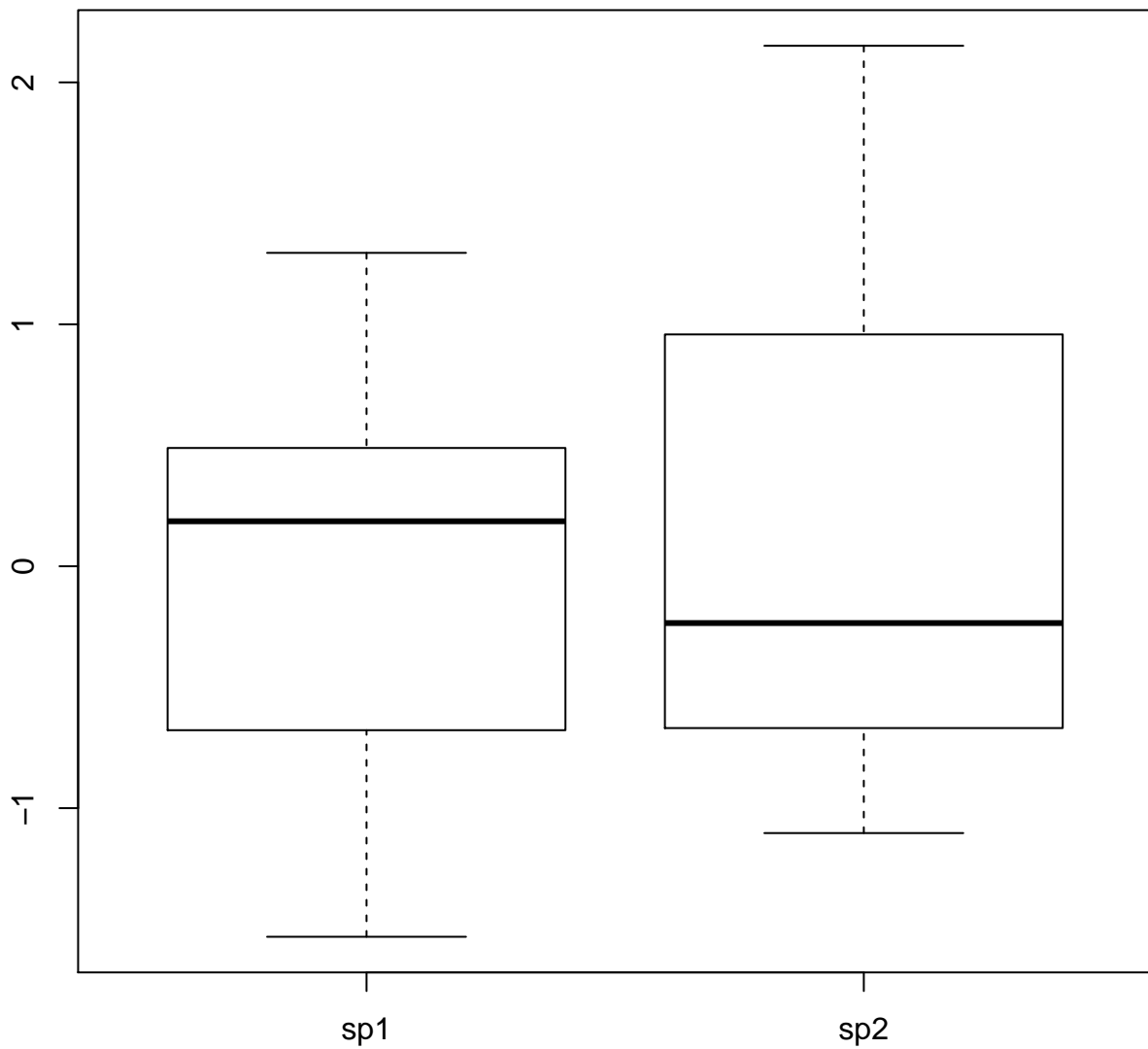

# PAL

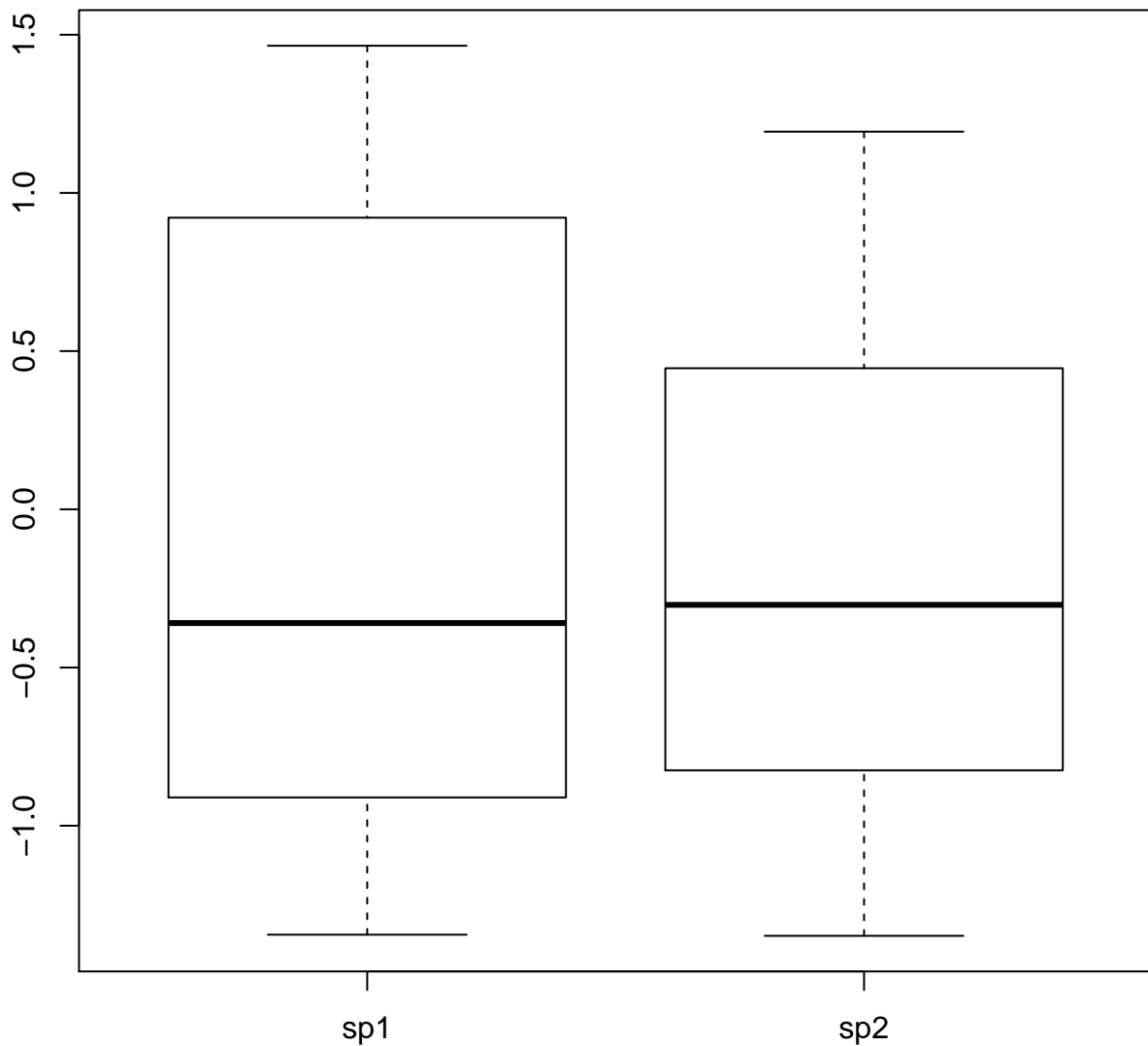

# PPL

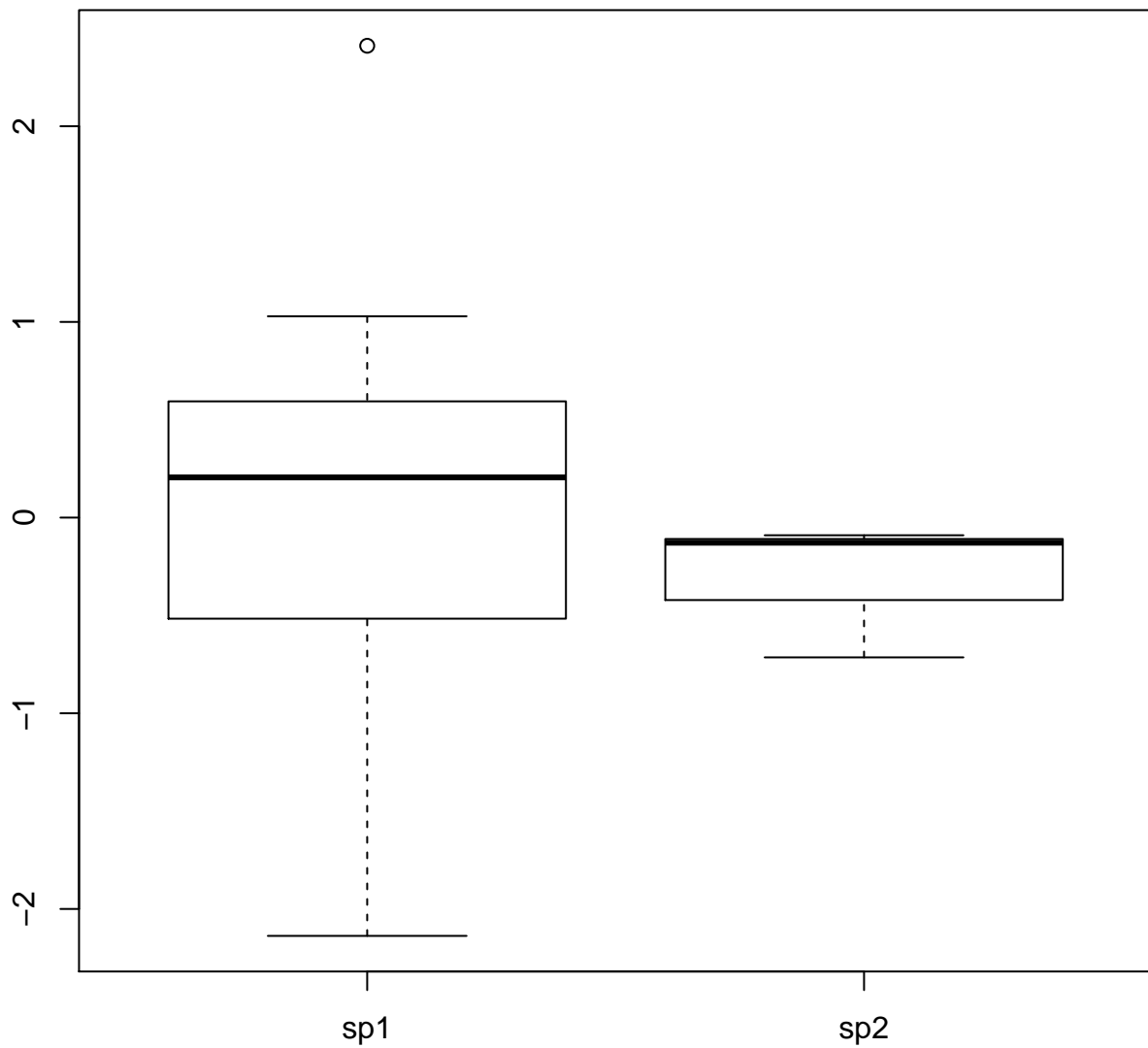

DFL

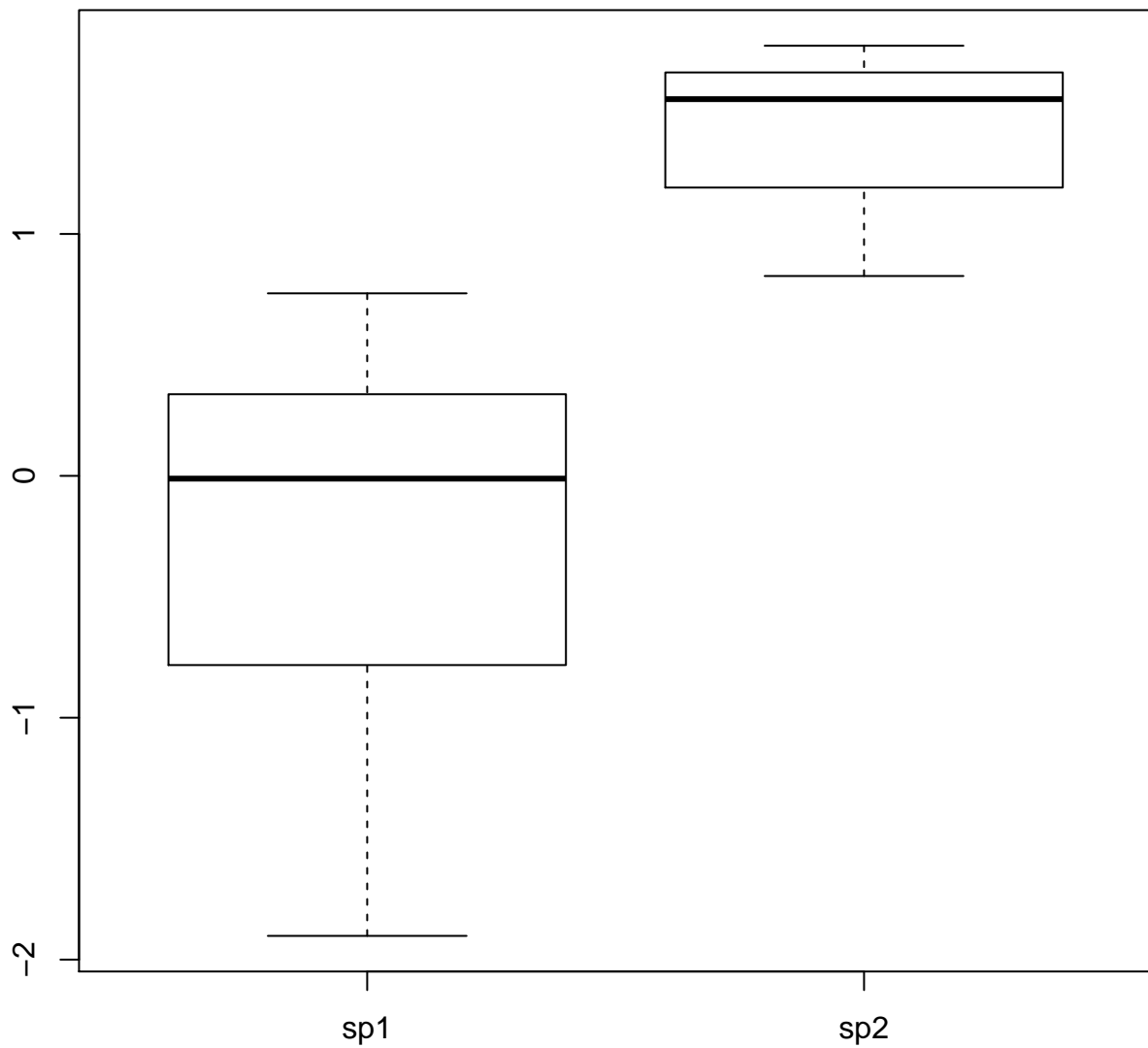

# AFL

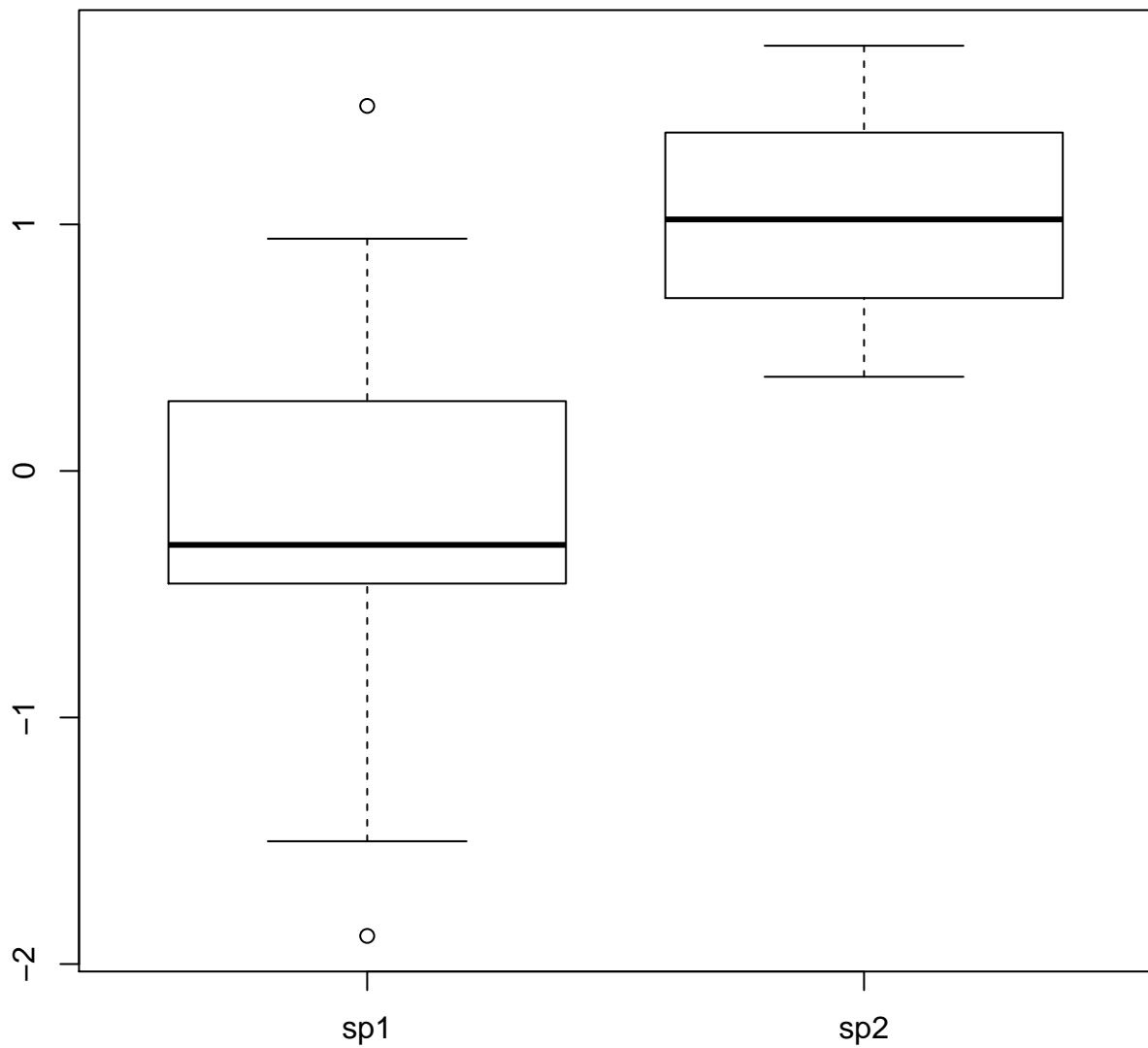

# PCL

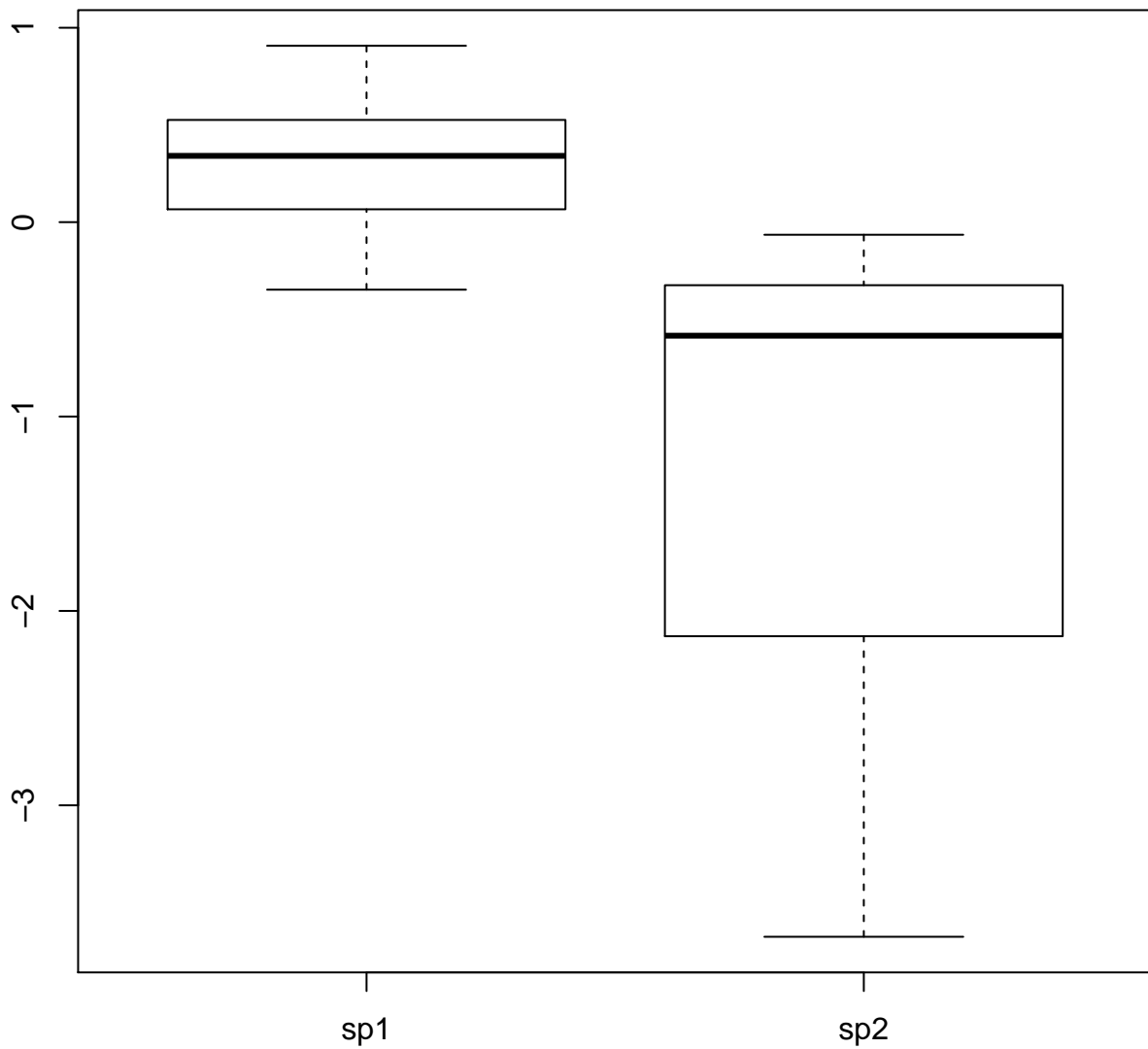

# PVL

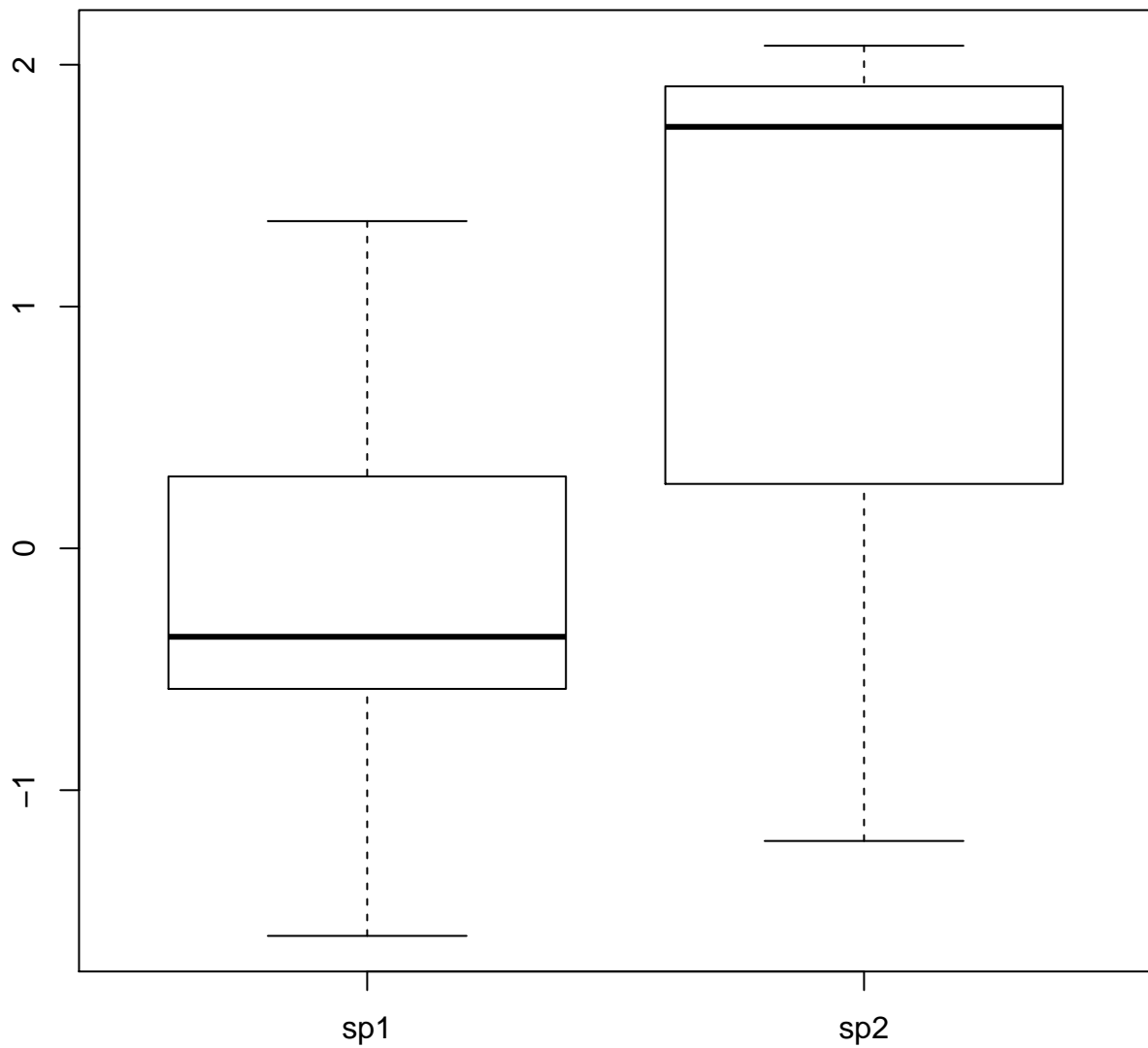

# PVS

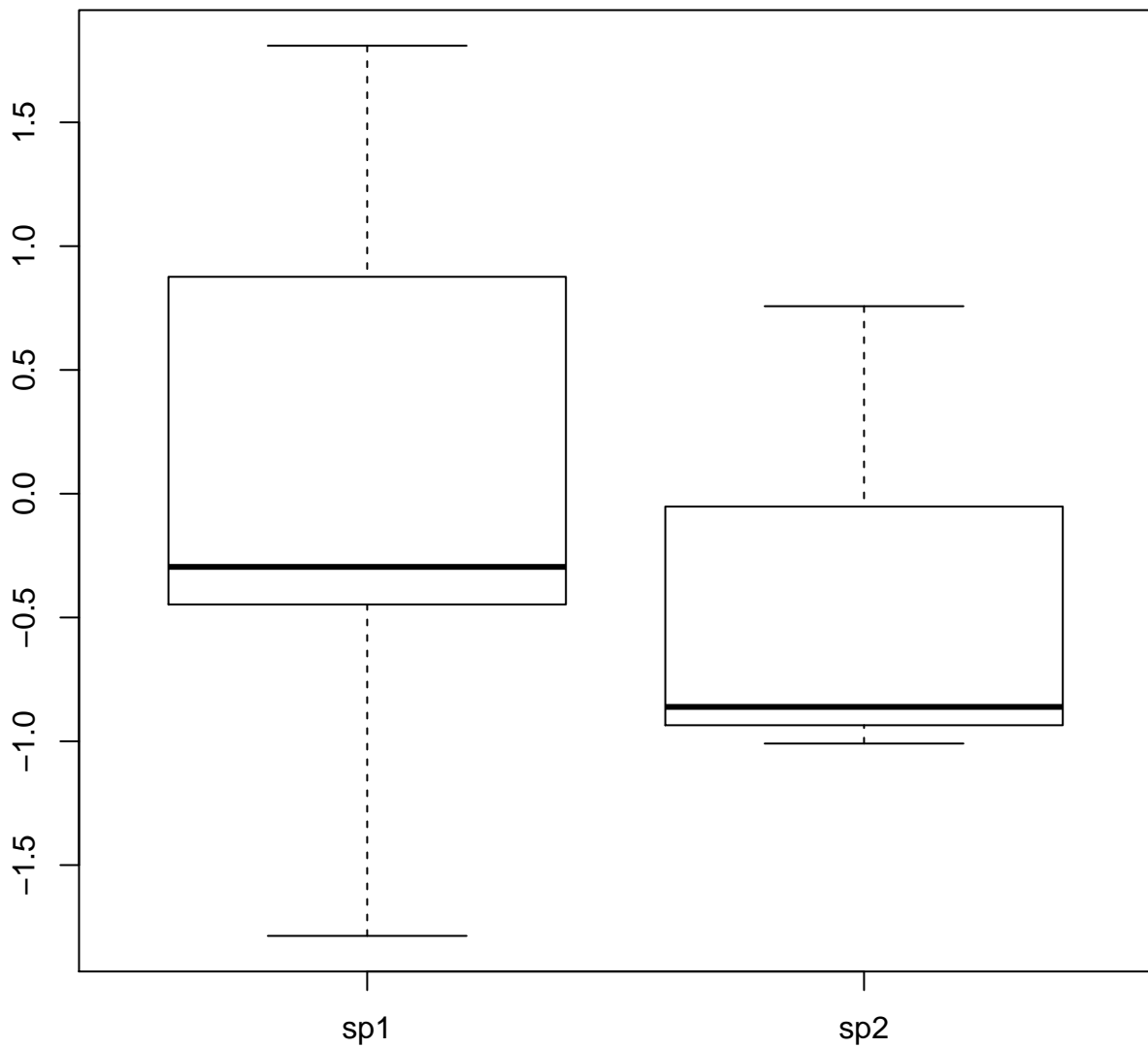

**DS1**

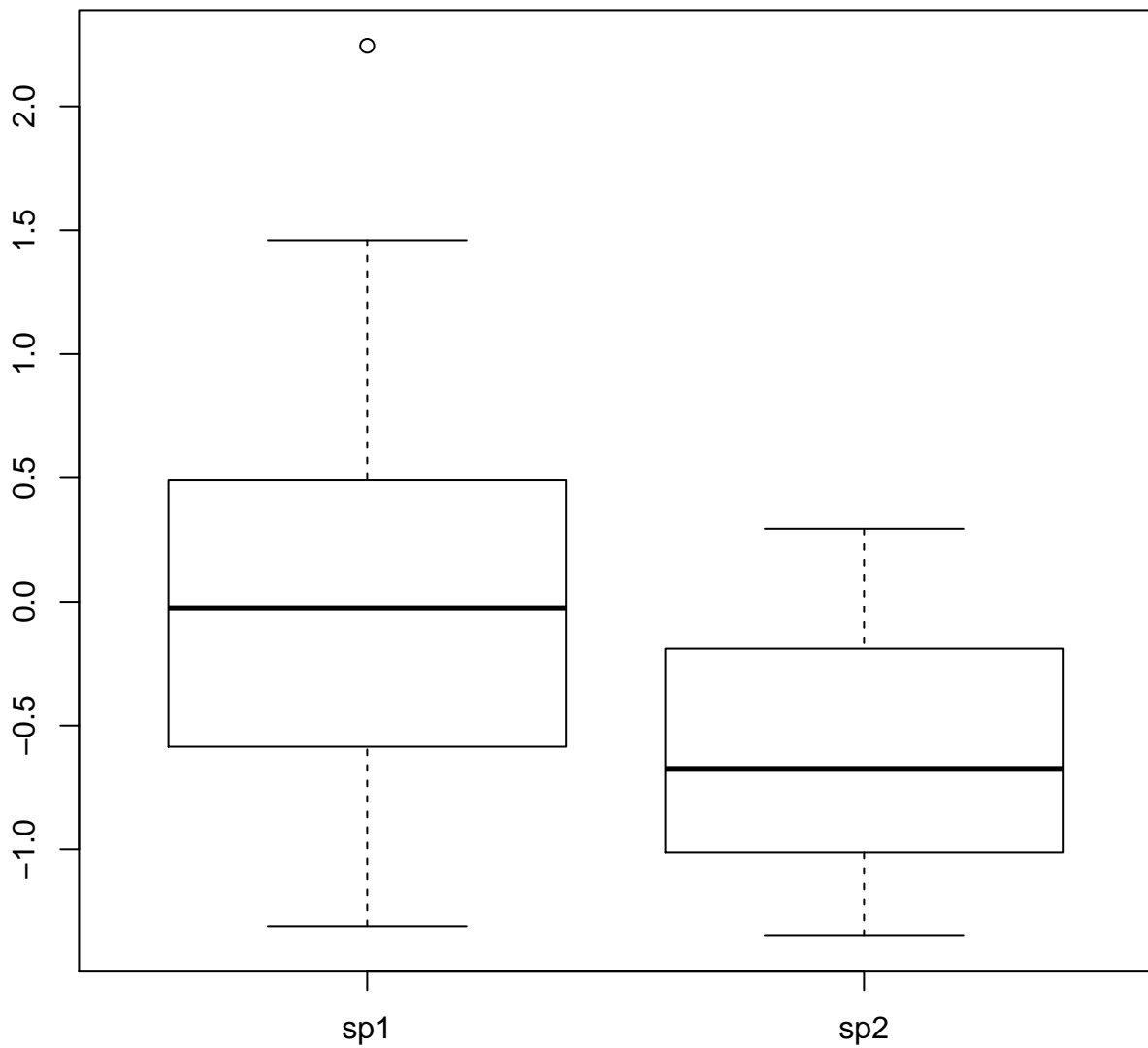

**DS2**

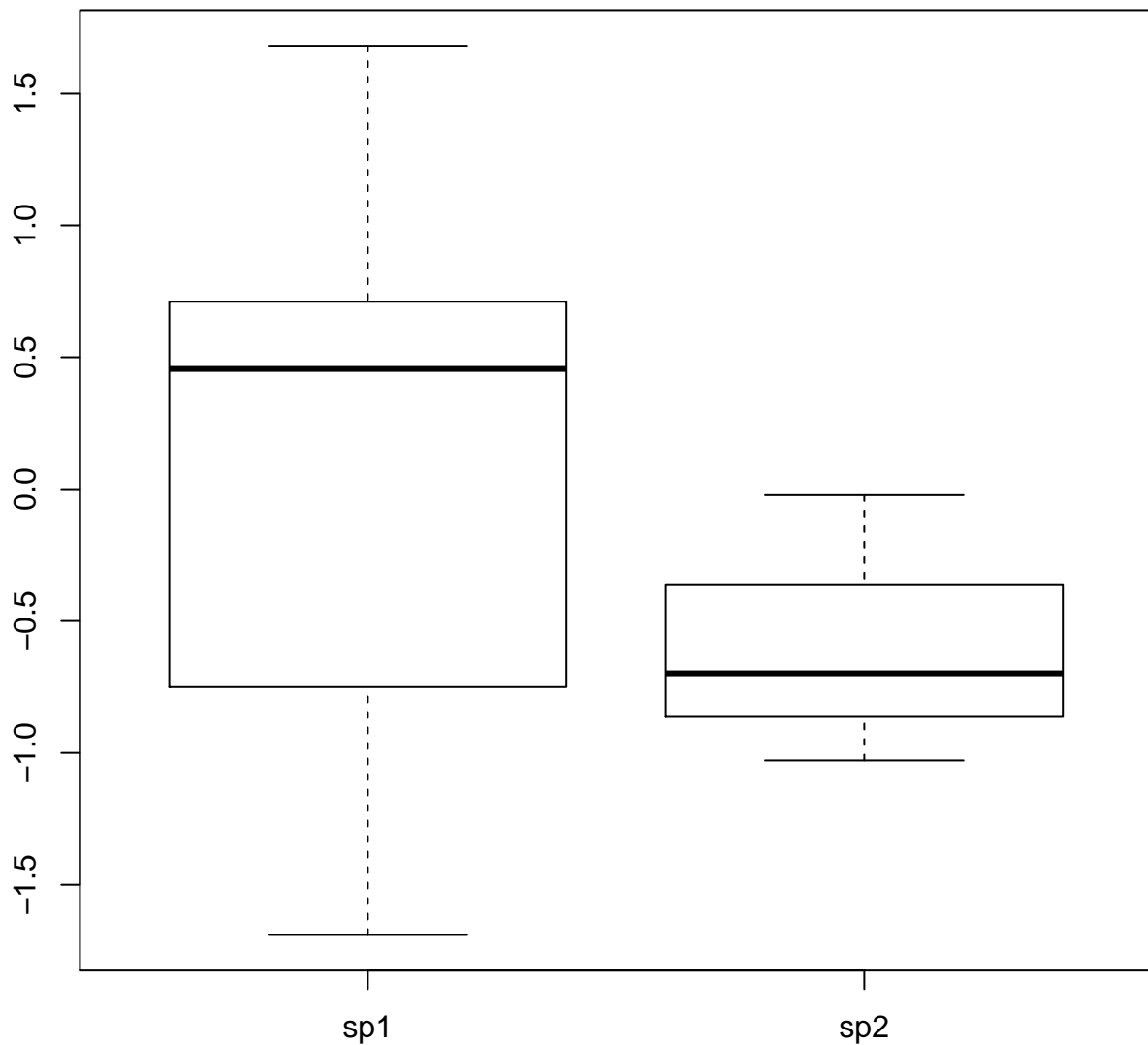

**DS3**

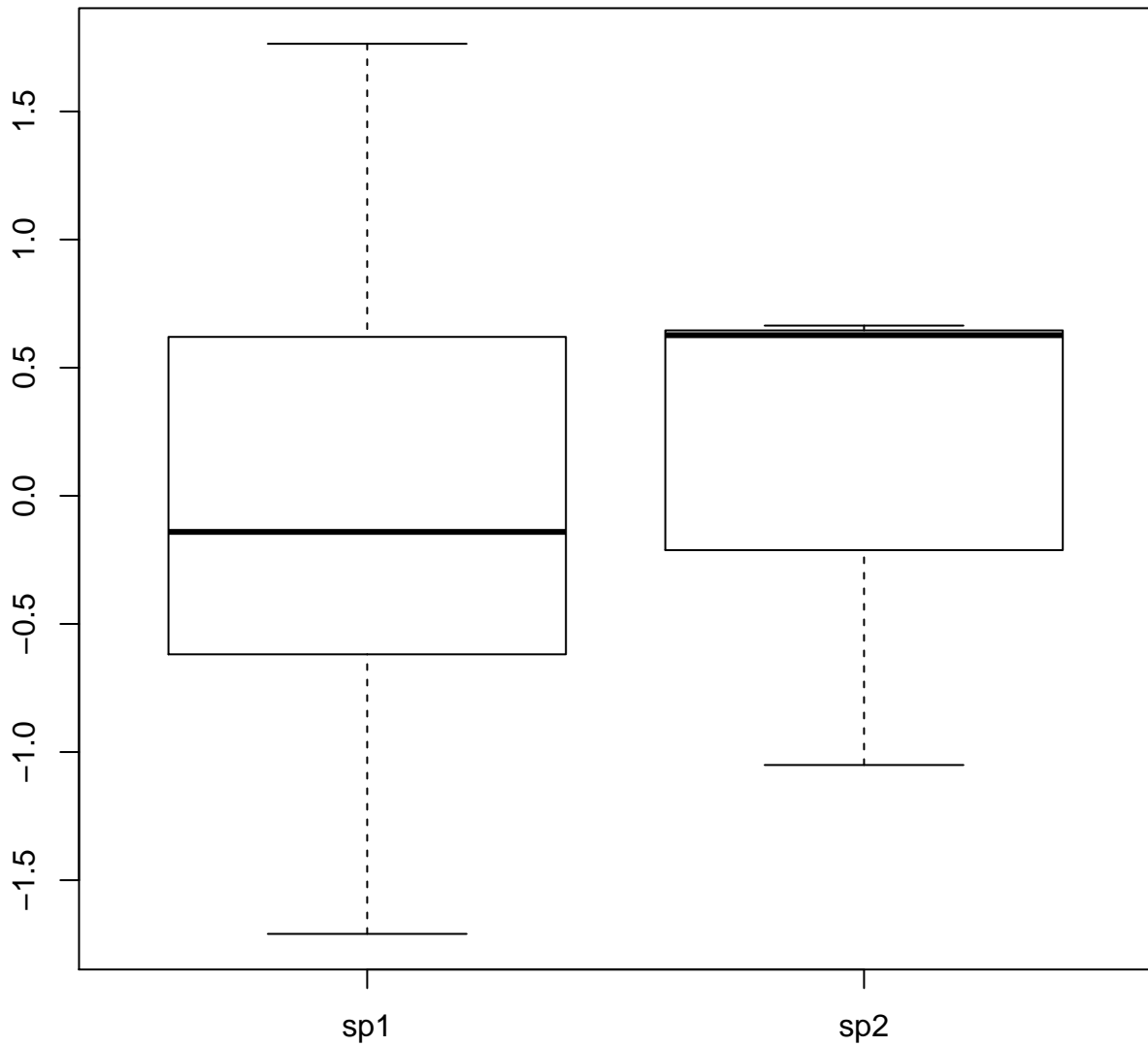

# DSL

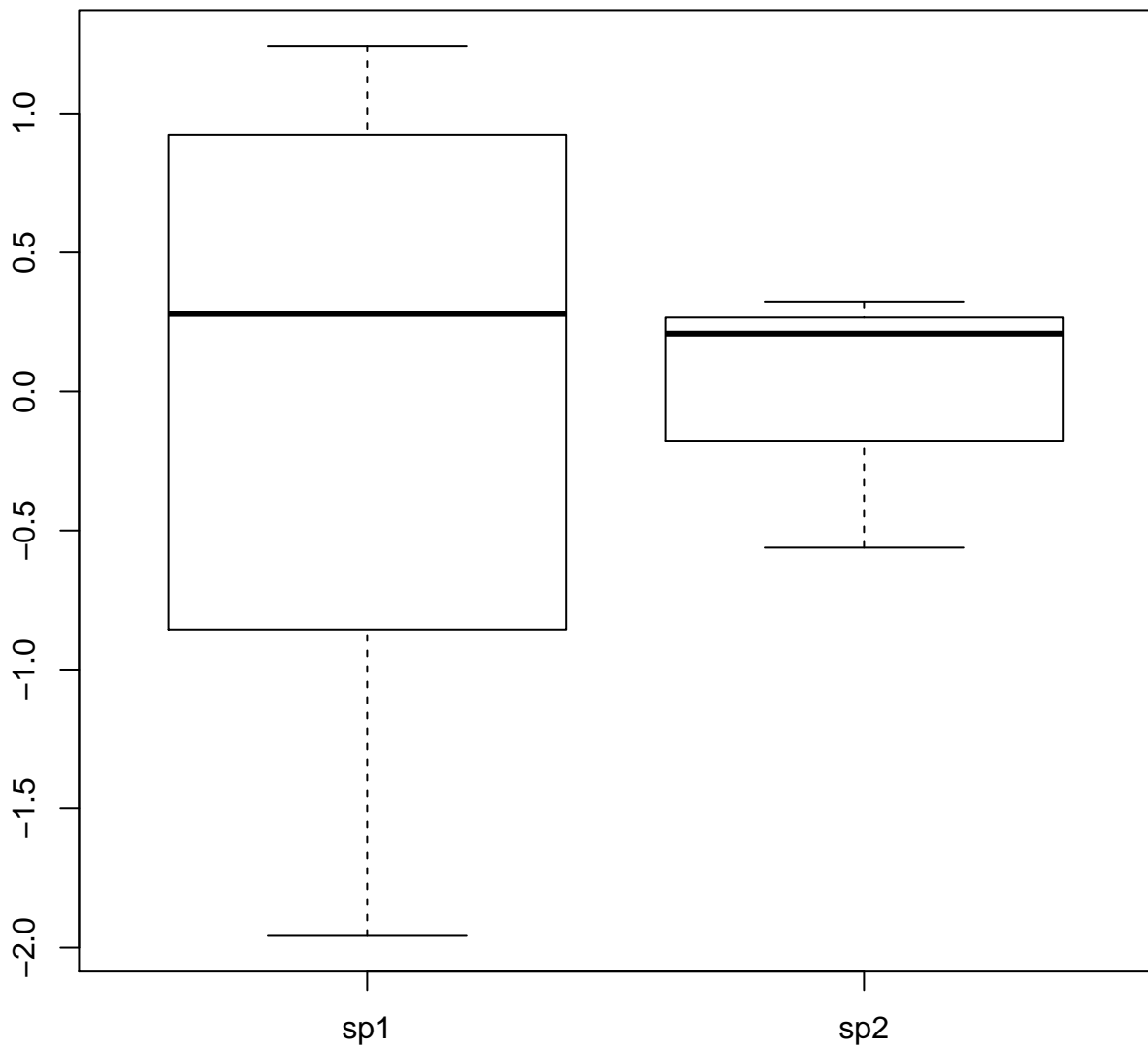

# DRL

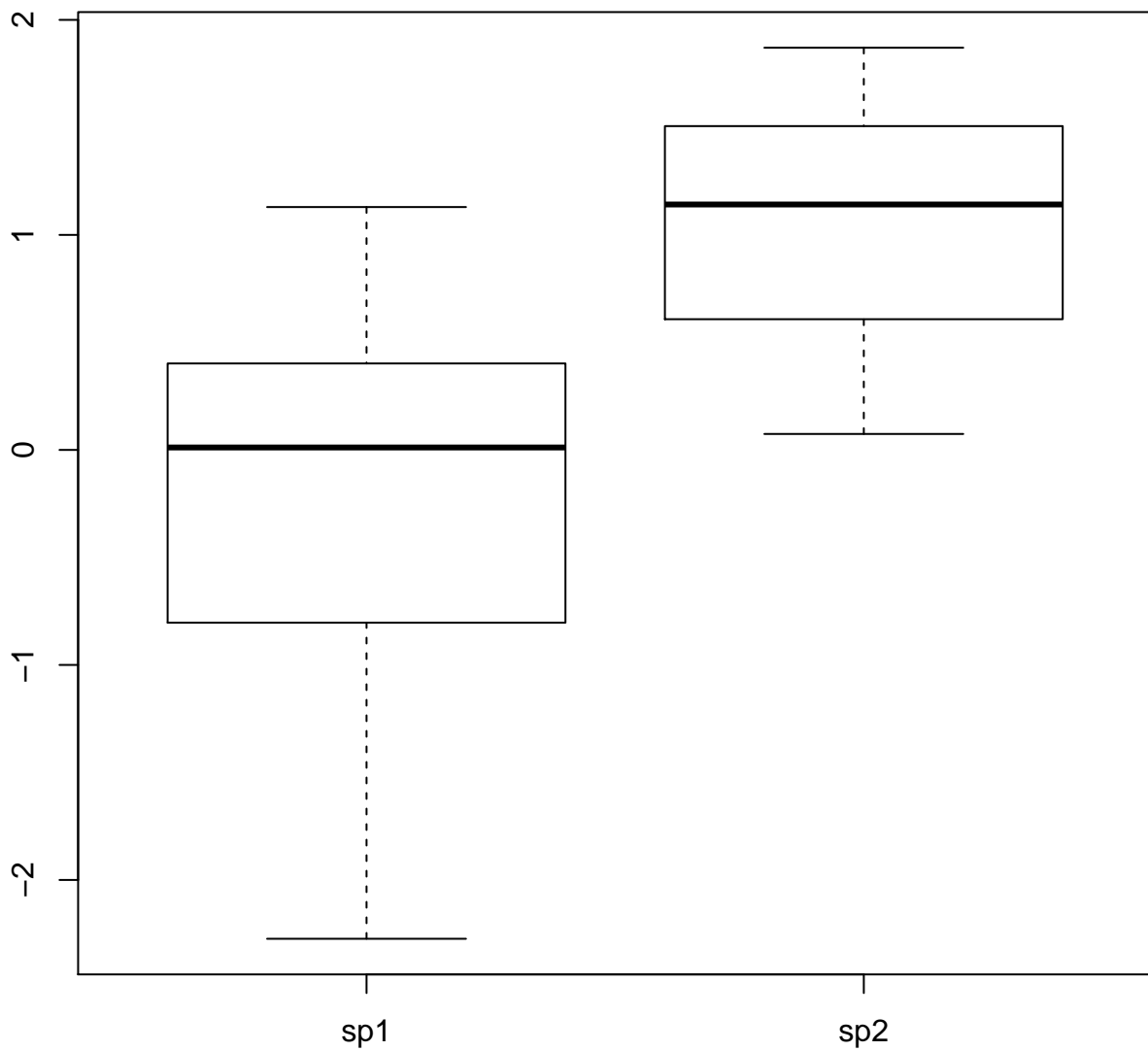

**AS1**

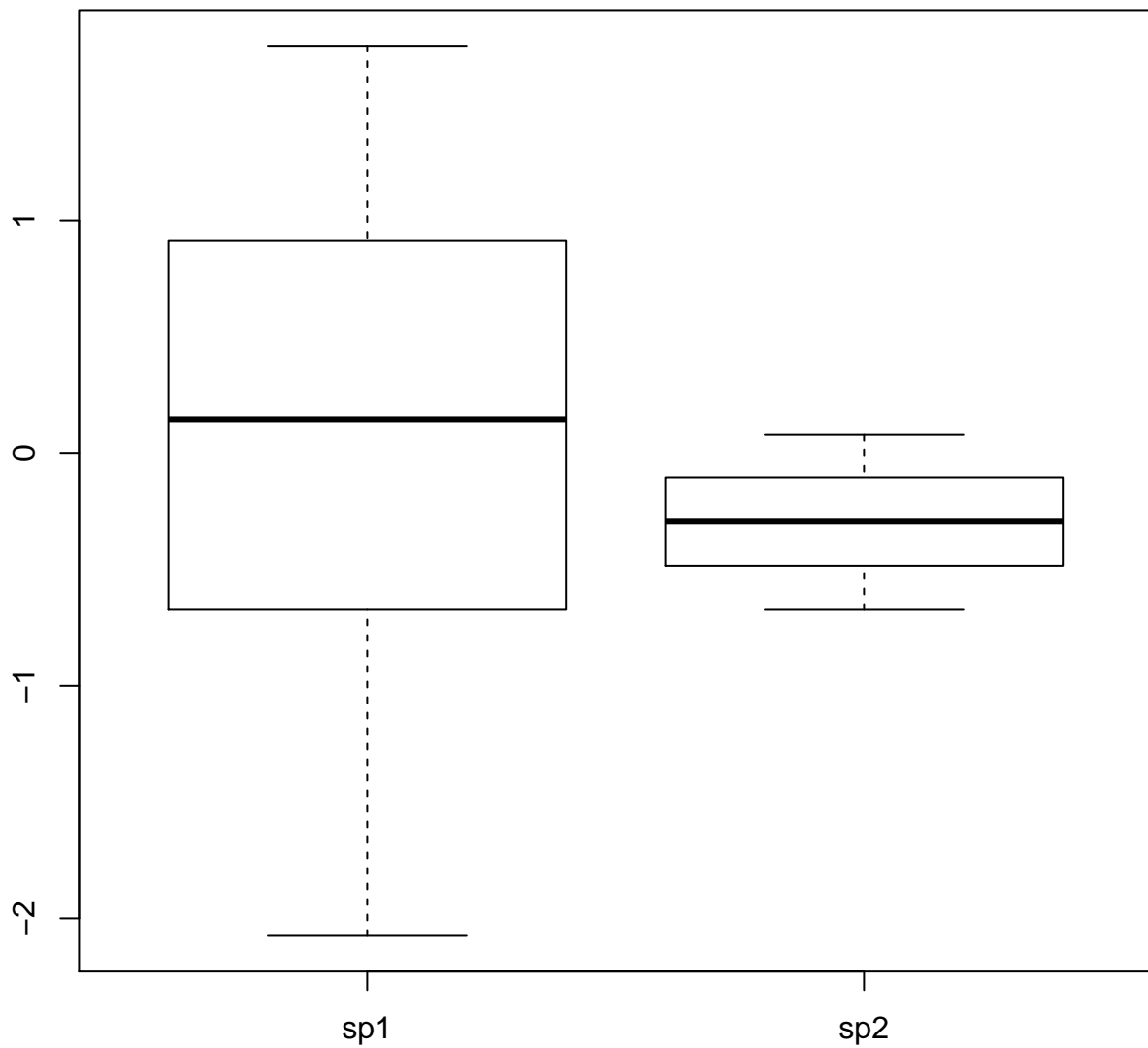

# AS2

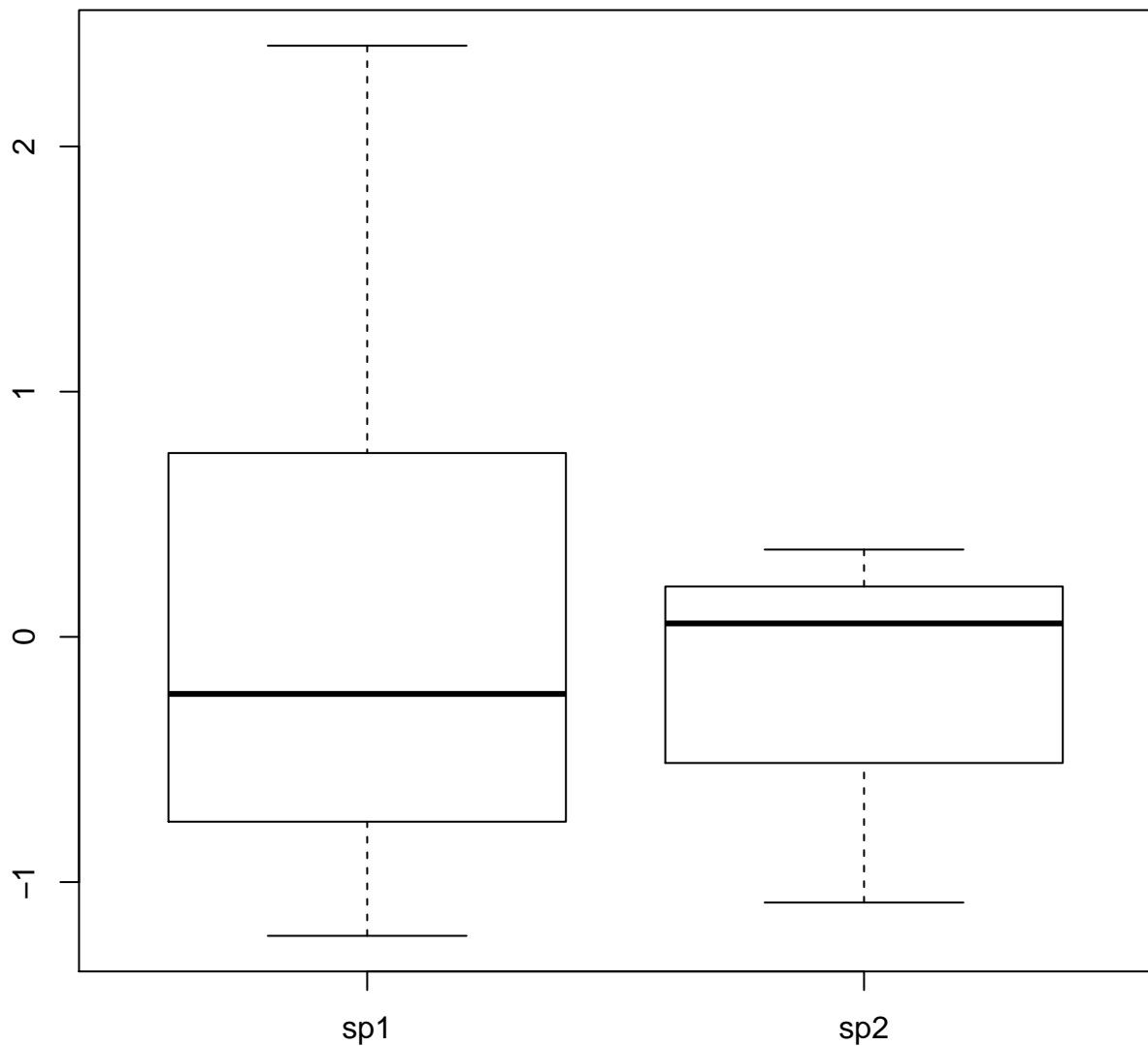

# ARL

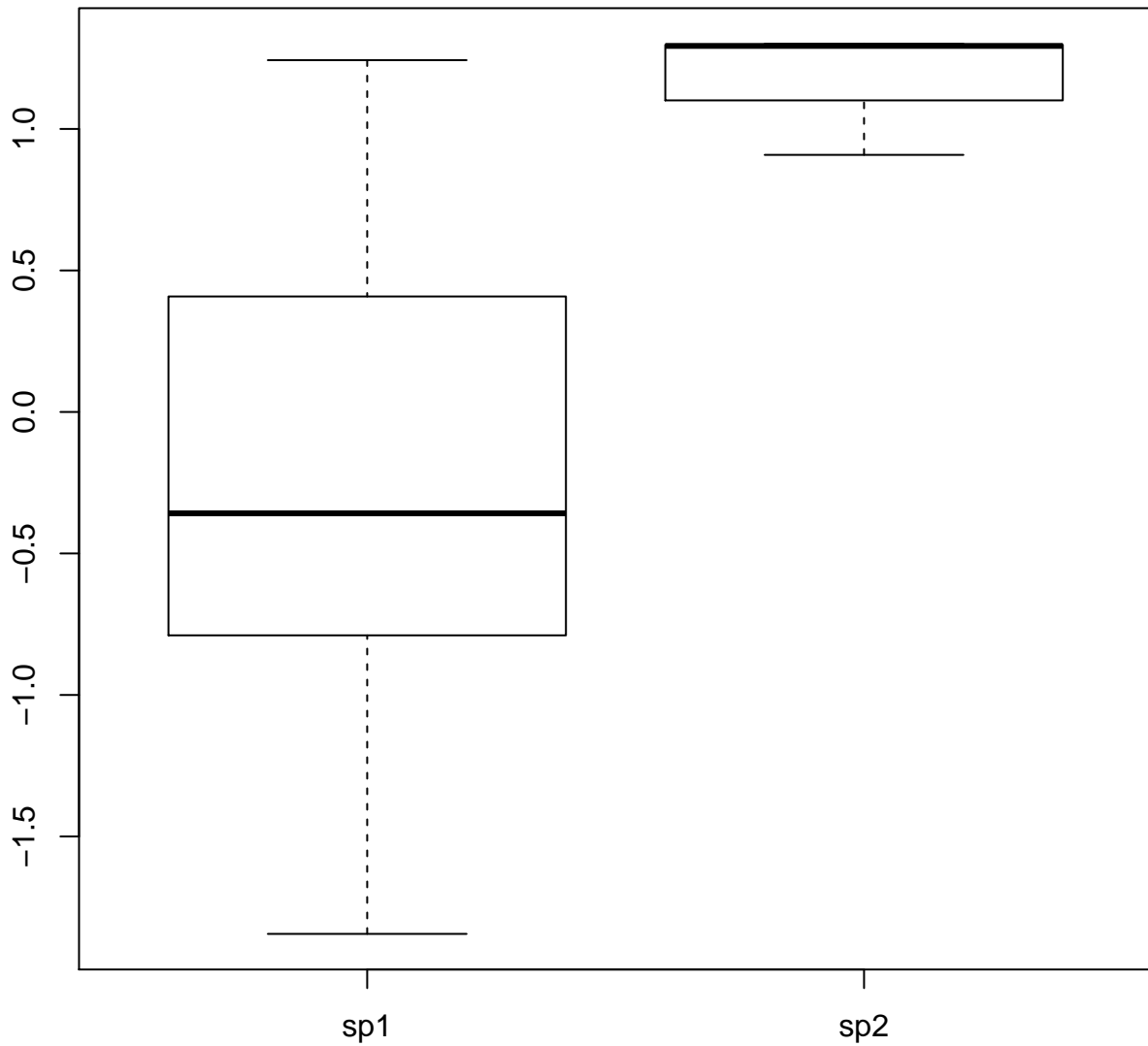

# CFL

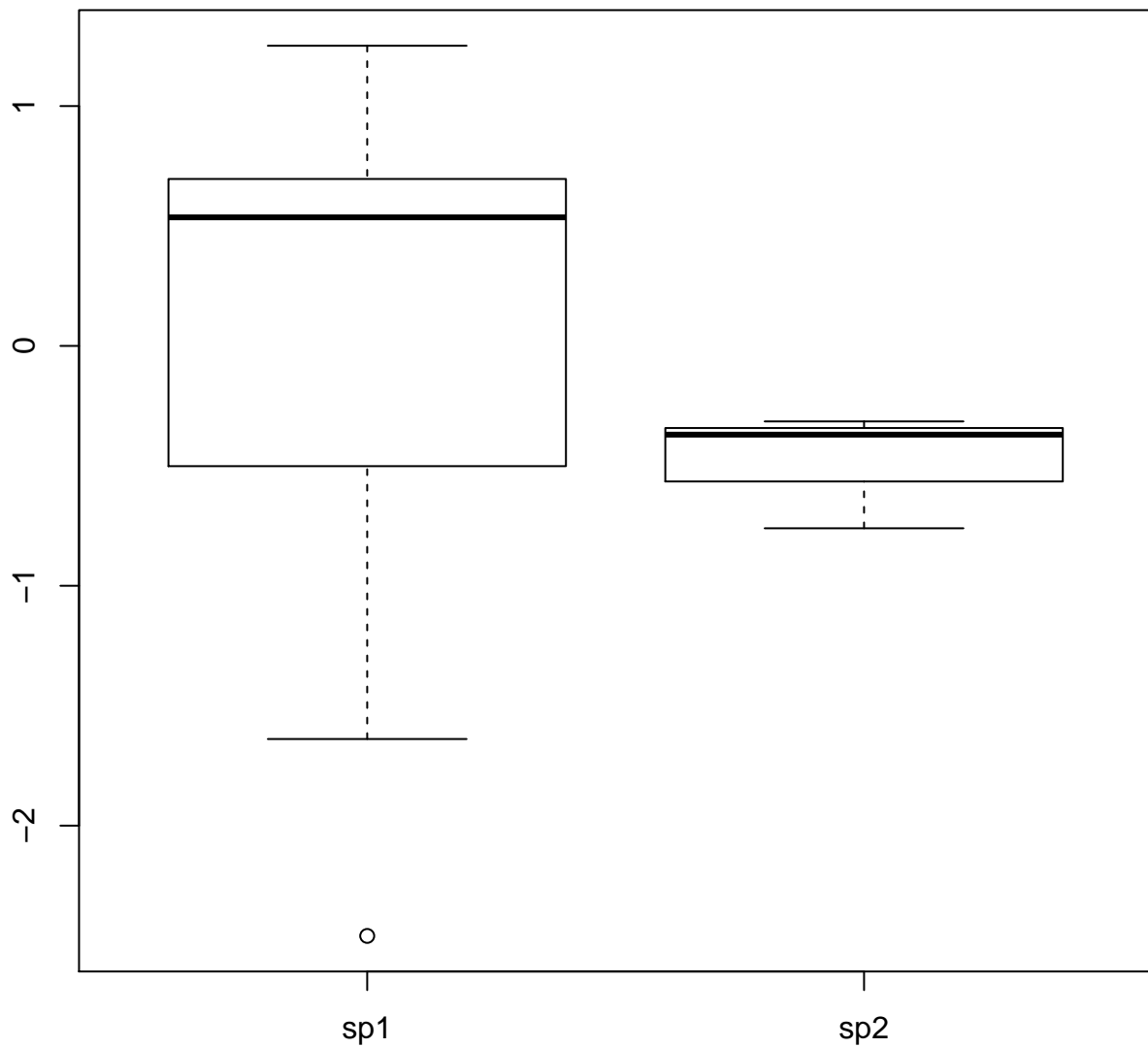

# B1W

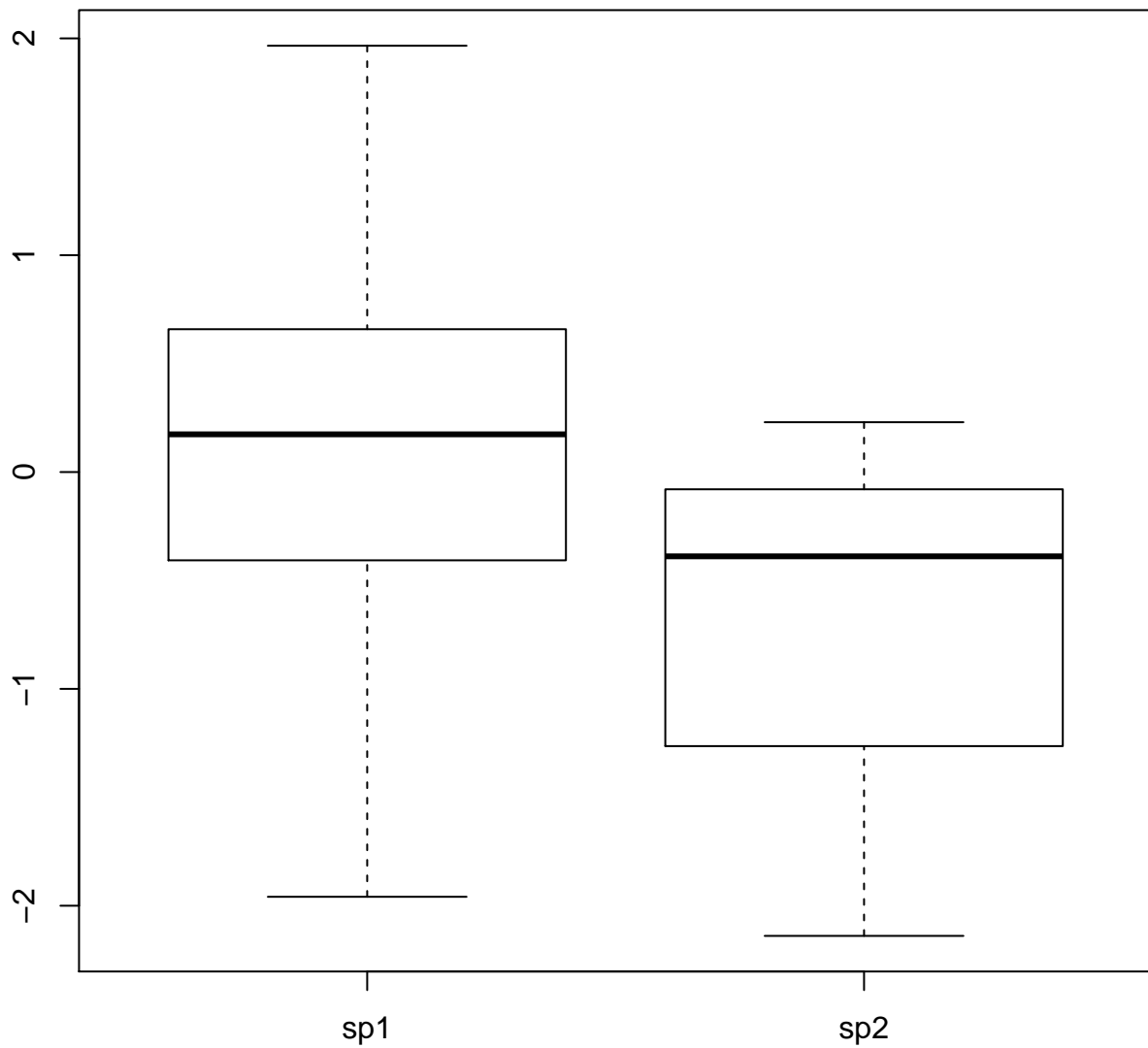

# B2W

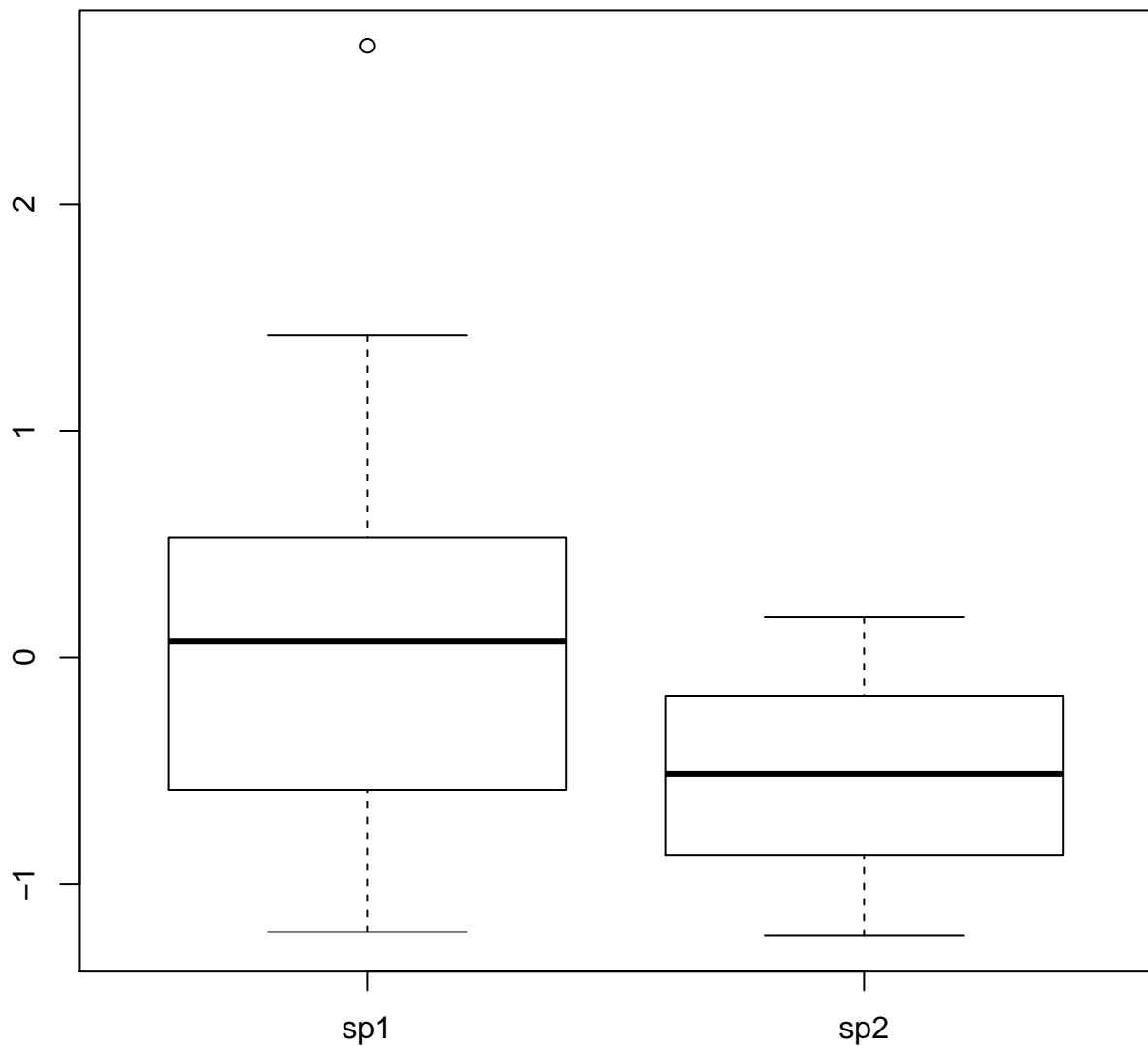

Supplement: Supplementary material 1 — Distribution of morphological characters taken individually for Amphiprionchrysopterus (species 1) and Amphiprionmaohiensis (species 2) [file zookeys-1244-225_article-141409__-s001.pdf]
